# Reconceptualizing Schizophrenia through a Patient-Oriented Perspective: a Systematic Review and Qualitative Meta-Synthesis

**DOI:** 10.1093/schbul/sbag122

**Published:** 2026-09-25

**Authors:** Sofia Francesca Aprile, Alessandro Rodolico, Djordje Basic, Kerem Böge, Markus Bühner, Christoph U Correll, Cristiano Cutuli, Peter Falkai, Gerhard Gründer, Alkomiet Hasan, Stefania Lanzafame, Josef Priller, Christian Nikolaus Schmitz, Maria Salvina Signorelli, Irene Bighelli, Filippo Caraci, Stefan Leucht

**Affiliations:** Department of Educational Sciences, Section of Psychology, University of Catania, 95124 Catania, Italy; Department of Psychiatry and Psychotherapy, TUM School of Medicine and Health, Technical University of Munich, Klinikum rechts der Isar, 81675 Munich, Germany; Department of Psychiatry and Psychotherapy, TUM School of Medicine and Health, Technical University of Munich, Klinikum rechts der Isar, 81675 Munich, Germany; German Center for Mental Health, DZPG, partner site München/Augsburg, 80336 Munich, Germany; Department of Clinical and Experimental Medicine, Psychiatry Unit, University of Catania, 95123 Catania, Italy; Department of Psychiatry and Psychotherapy, TUM School of Medicine and Health, Technical University of Munich, Klinikum rechts der Isar, 81675 Munich, Germany; Department of Clinical, Neuro and Developmental Psychology, Amsterdam Public Health Research Institute, Vrije Universiteit Amsterdam, 1081 BT Amsterdam, the Netherlands; German Center for Mental Health, DZPG, partner site Berlin, 10099 Berlin, Germany; Department of Psychiatry and Psychotherapy, Campus Benjamin Franklin, Charité—Universitätsmedizin Berlin, and Freie Universität Berlin, Humboldt-Universität zu Berlin, and Berlin Institute of Health, 12203 Berlin, Germany; Medical University Brandenburg—Theodor Fontane, 16816 Neuruppin, Germany; Department of Psychology, Ludwig Maximilian University of Munich, 80802 Munich, Germany; Department of Child and Adolescent Psychiatry, Charité—Universitätsmedizin Berlin, 13353 Berlin, Germany; Department of Psychiatry and Molecular Medicine, Donald and Barbara Zucker School of Medicine at Hofstra/Northwell, Hempstead, NY 11549, United States; Department of Psychiatry, Zucker Hillside Hospital, Northwell Health System, Glen Oaks, NY 11004, United States; Department of Clinical and Experimental Medicine, Psychiatry Unit, University of Catania, 95123 Catania, Italy; Department of Psychiatry, Munich University Hospital, 80336 Munich, Germany; Max-Planck-Institute of Psychiatry, 80804 Munich, Germany; Department of Molecular Neuroimaging, Central Institute of Mental Health, Medical Faculty Mannheim, University of Heidelberg, 68159 Mannheim, Germany; German Center for Mental Health, DZPG, partner site München/Augsburg, 80336 Munich, Germany; Department of Psychiatry, Psychosomatics, and Psychotherapy, University of Augsburg, 86156 Augsburg, Germany; Department of Clinical and Experimental Medicine, Psychiatry Unit, University of Catania, 95123 Catania, Italy; Department of Psychiatry and Psychotherapy, TUM School of Medicine and Health, Technical University of Munich, Klinikum rechts der Isar, 81675 Munich, Germany; German Center for Mental Health, DZPG, partner site München/Augsburg, 80336 Munich, Germany; Edinburgh Medical School, UK Dementia Research Institute at the University of Edinburgh, Chancellor’s Building, Edinburgh EH16 4SB, United Kingdom; Centre for Clinical Brain Sciences, University of Edinburgh, Chancellor’s Building, Edinburgh EH16 4SB, United Kingdom; Neuropsychiatry and Laboratory of Molecular Psychiatry, Charité—Universitätsmedizin Berlin and DZNE, 10117 Berlin, Germany; Department of Molecular Neuroimaging, Central Institute of Mental Health, Medical Faculty Mannheim, University of Heidelberg, 68159 Mannheim, Germany; Central Institute of Mental Health, Department of Psychiatry, Medical Faculty Mannheim, University of Heidelberg, 68159 Mannheim, Germany; Department of Clinical and Experimental Medicine, Psychiatry Unit, University of Catania, 95123 Catania, Italy; Oasi Research Institute—IRCCS, Unit of Neuropharmacology and Translational Neurosciences, 94018 Troina, Italy; Department of Psychiatry and Psychotherapy, TUM School of Medicine and Health, Technical University of Munich, Klinikum rechts der Isar, 81675 Munich, Germany; German Center for Mental Health, DZPG, partner site München/Augsburg, 80336 Munich, Germany; Oasi Research Institute—IRCCS, Unit of Neuropharmacology and Translational Neurosciences, 94018 Troina, Italy; Department of Drug and Health Sciences, University of Catania, 95123 Catania, Italy; Department of Psychiatry and Psychotherapy, TUM School of Medicine and Health, Technical University of Munich, Klinikum rechts der Isar, 81675 Munich, Germany; German Center for Mental Health, DZPG, partner site München/Augsburg, 80336 Munich, Germany

**Keywords:** lived experience, qualitative meta-synthesis, stigma, fear, cognitive impairment, person-centered care

## Abstract

**Background and Hypothesis:**

Schizophrenia spectrum disorders are typically described using positive and negative symptoms, yet standard diagnostic systems may not reflect the full range of how individuals experience the disorder. We hypothesized that synthesizing first-person accounts would yield a broader, clinically useful symptom framework that better represents lived experience.

**Study Design:**

We performed a systematic review and qualitative meta-synthesis following Enhancing Transparency in Reporting the Synthesis of Qualitative Research guidelines (PROSPERO: CRD420251069446). PubMed, PsycINFO, and Scopus were searched up to January 2026. Eligible studies used qualitative or mixed methods and reported first-person accounts from individuals with schizophrenia spectrum disorders. We included 52 peer-reviewed studies (2856 participants). We conducted two thematic syntheses: (1) general illness experience and (2) symptom experience organized using the Manual for Assessment and Documentation of Psychopathology in Psychiatry framework.

**Study Results:**

Across illness experience, five themes emerged: pervasive effects on physical, psychological, social, and relational life; stigma and isolation; interactions with medication, clinicians, and services; disruption and fragmentation of self, reality, and meaning; and coping strategies, including the protective role of social support. Across symptom experience, fear recurred across psychotic and non-psychotic domains, and participants often described psychosis as a transformed experience of reality rather than discrete symptoms. Accounts spanned disturbances in consciousness, perception, thinking, cognition, affect, and behavior.

**Conclusions:**

Lived experience highlights marked heterogeneity that extends beyond traditional symptom dichotomies and is only partly captured by current diagnostic frameworks. Incorporating patient-reported perspectives may support more nuanced, person-centered assessment and evaluation of treatment response.

## Introduction

Schizophrenia spectrum disorders (SSDs) profoundly impair functioning and quality of life.^1^ According to the Diagnostic and Statistical Manual of Mental Disorders, Fifth Edition, Text Revision (DSM-5-TR), the diagnosis requires the presence of at least two core symptoms, such as delusions, hallucinations, disorganized speech, grossly disorganized or catatonic behavior, and negative symptoms, persisting for a significant portion of 1 month, with continuous signs of disturbance for at least 6 months.^2^ Similarly, the International Classification of Diseases, 11th Revision (ICD-11) defines SSDs as marked by disturbances in thought, perception, self-experience, cognition, and volition that result in significant impairment in personal, social, and occupational functioning, but expects the symptoms to be present for at least six months.^3^ The Chinese Classification of Mental Disorders, third edition (CCMD-3), characterizes SSDs by prominent disturbances in thinking, affect, and volition, lasting for at least 1 month and often accompanied by impaired social functioning, with diagnostic emphasis placed on persistent thought disorder and incongruent affect.^4^ However, while the diagnostic criteria provide a crucial framework for establishing a common clinical language, they often appear reductive when compared with the multifaceted nature of complex and heterogeneous neuropsychiatric disorders such as SSDs. One way of assessing the broad symptoms of SSDs includes the widely used Positive and Negative Syndrome Scale (PANSS). Factor analyses of the PANSS have proposed a five-factor structure, grouping symptoms into positive, negative, disorganized, excited, and depressive dimensions.^5^^,^^6^ While this model captures important aspects of the disorder, it does not fully account for other core features, such as cognitive impairment, which represents a distinct yet interconnected symptomatic dimension.^7^ Similarly, affective disturbances, particularly depression and anxiety, are highly prevalent and strongly associated with neurocognitive deficits in domains such as executive functioning, attention, memory, and social cognition.^8^ Taken together, these frameworks highlight complementary but only partial perspectives, underscoring that a comprehensive conceptualization of SSDs’ symptomatology is still lacking. Multiple symptom dimensions have been identified in SSDs, reflecting its wide heterogeneity in clinical manifestations; however, reaching a definition that is both concise and clinically usable, while still capturing this complexity, remains an ongoing challenge. We hypothesize that grounding the definition of this complex neuropsychiatric disorder in subjects’ lived experience may provide a more comprehensive and meaningful understanding. A patient-oriented approach is gaining increasing recognition in psychiatry, largely due to the inherent challenges of establishing diagnoses with absolute certainty as well as in clinical psychopharmacology to better assess the impact of the antipsychotics treatment.^9^^,^^10^ Recently, a patient-centered framework grounded in the fulfilment of individual resources and goals has been proposed to foster multidisciplinary care and to support more personalized treatment strategies.^11^ First-person accounts have also been increasingly recognized as an important source of knowledge for understanding psychosis. Phenomenological psychiatry has long highlighted disturbances of self-experience and meaning-making as central features of schizophrenia,^12^^,^^13^ while recent work co-produced with experts by experience has shown how lived experience may both complement and challenge standard symptom–based frameworks.^10^ In this perspective, patient-reported experiences are not only descriptive, but may also reveal dimensions insufficiently captured by current diagnostic systems. The present study was designed to provide a patient-centered perspective on schizophrenia that extends beyond conventional diagnostic descriptions, while maintaining clinical interpretability. Given that traditional diagnostic frameworks may not fully capture the complexity of schizophrenia, the aim of the present study was to synthesize individuals’ lived experiences of symptoms in SSDs.

## Methods

### Search Strategy

This systematic review was conducted in accordance with the ENTREQ framework (Supplementary materials, Appendix SE).^14^ A comprehensive search was performed in PubMed, PsycINFO, and Scopus, with the last search conducted on January 12, 2026. No restrictions on language or publication date were applied. The search strategy combined Medical Subject Headings and free-text keywords; an example with full query strings is available in the supplementary materials (Appendix SA). Reference lists of all included studies were also screened manually by two independent reviewers (S.F.A. and C.C.) to identify additional relevant publications. A full list of excluded studies, with reasons for exclusion, is provided in Appendix SB. The review protocol was developed prospectively and registered in PROSPERO (ID: CRD420251069446).

### Eligibility Criteria

The following inclusion criteria were applied:

Population: studies enrolling participants diagnosed with SSDs (schizophrenia, schizoaffective disorder, schizophreniform disorder, delusional disorder, brief psychotic disorder, drug-induced psychosis) according to the following established diagnostic systems: DSM-III, DSM-III-R, DSM-IV-TR, DSM-5, ICD-10, ICD-11, CCMD-3. In studies including mixed diagnostic groups, qualitative data had to be clearly attributable to individuals with SSDs and extracted independently. When differentiation was not possible, studies were included only if subjects with SSDs diagnoses represented at least 80% of the sample.
Study design: original qualitative or mixed-methods studies were eligible. Examples of qualitative methodologies include in-depth interviews, focus groups, and ethnographic approaches. In mixed-methods studies, qualitative findings had to be explicitly reported and clearly distinguishable.
Phenomenon of interest: studies exploring subjects’ lived experiences of SSDs symptoms from a first-person perspective were included. Studies that also investigated caregivers’ or healthcare professionals’ perspectives were eligible only if subjects’ data could be clearly separated.
Publication type: only original research articles were included.
Setting: studies conducted in any setting (eg, inpatient, outpatient, or community-based services) were considered.

### Data Extraction

**Figure 1:**
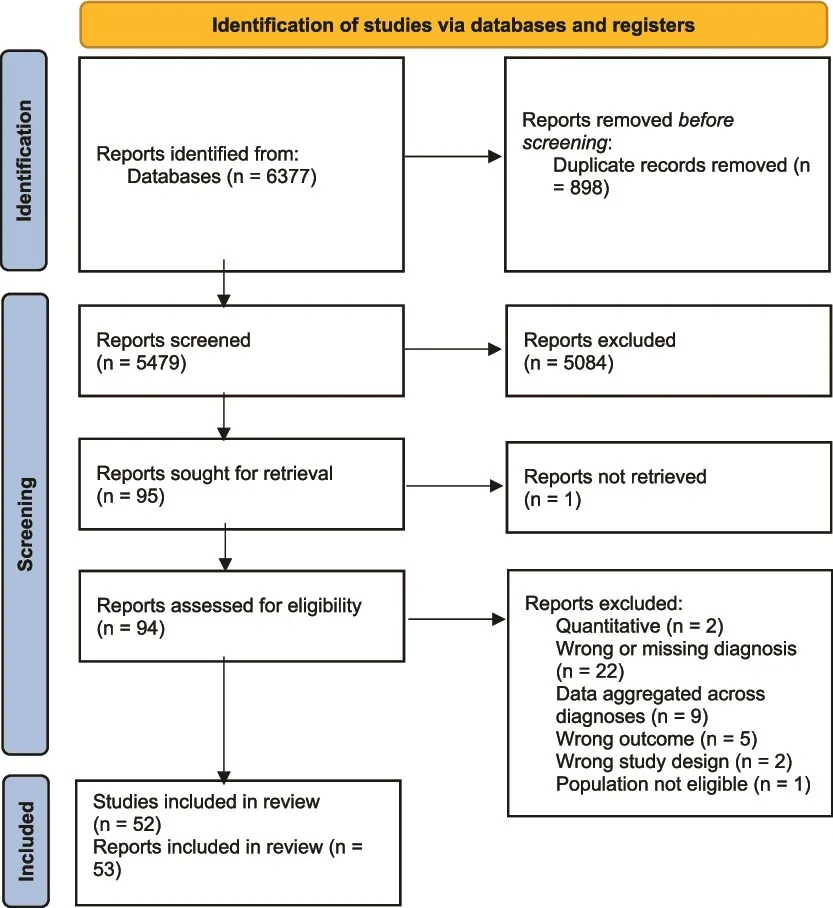
PRISMA Flow Diagram Illustrating the Study Selection Process.

The screening and selection process was carried out independently by two researchers (S.F.A. and C.C.) and is illustrated in the PRISMA flowchart (Figure 1). Discrepancies were resolved through discussion with a senior researcher (A.R.), when necessary. In line with ENTREQ guidelines for qualitative evidence synthesis, the quality of included studies was assessed using the Critical Appraisal Skills Programme (CASP) Qualitative Checklist,^15^ which comprises 10 items. Each study was appraised independently by two reviewers (S.F.A. and S.La.), and the CASP scores are summarized in Table 1. Detailed item–level assessments (yes/no responses) are provided in the supplementary materials for full transparency. Qualitative data were extracted mainly from the Results sections of the included studies, including any tables or supplementary material referenced therein, to ensure the preservation of participants’ contextual accounts. Efforts were made to contact study authors to obtain missing relevant data for the thematic synthesis.

**Table 1:** Perception of SSDs from Subjects’ Perspective

Theme	Sub-theme	Example quote
(a) Burden and impact of illness	Social impairment	“When you end up in the hospital system and all that, you do lose track of friends from before”^23^
	Physical and cognitive impairment	“I find that I’m so tired”^40^
	Disruption of life roles	“I gave up on parenthood a long time ago”^40^
	Motivational loss	“[...] just the disease making it impossible to set your goals”^41^
(b) Isolation, stigma, desire to belong	Stigma and shame	“Say that I’ve got depression rather than schizophrenia because it sounds better”^23^
	Damaged self-image	“I’m fairly useless...weak...”^42^
	Distrust and broken dialog	“In the hospital [...] they do not let you express your thinking”^43^
	Emotional disconnection	“You’re always sort of like on the edge or alone in a crowd, that sort of thing”^41^
(c) Relationship with drug, clinicians and health care system	Ambivalence toward drugs	“Everyone would not want to be on medication, but [...] it keeps us out of hospital”^44^
	Experience of coercion	“They put you in a chemical straight jacket”^25^
	Negotiating agency	“I agreed to go along voluntarily, without violence”^25^
	Lack of information	“The doctors are so busy nowadays that they don’t really have time to talk”^40^
(d) Disruption of self, reality and narrative reconstruction	Loss of engagement	“Simply didn’t conceive the connections”^45^
	Fragmentation of self	“I feel like I’m broken [...] everything is broken”^46^
	Altered reality	“Everything I thought was real [...] none of this was even real”^47^
	Struggle in meaning-making	“You cannot put it into words, the words are missing to say how it feels”^48^
	Narrative reconstruction and existential reframing	“It’s important to see yourself as a person first [...] people with schizophrenia have the same desires as other people”^40^
(e) Support and coping strategies	Social support	“Having people in your life investing in you. That’s certainly what helps me.”^41^
	Functional coping strategies	“Well I’d talk with somebody about it. Helped quite a bit.”^49^
	Dysfunctional coping strategies	“Showing anger was one way of, I don’t know asking for help.”^50^

### Data Analysis

Coding and data analysis were conducted by S.F.A. and A.R. Data were first separated into two categories: (1) quotations describing the general illness experience without reference to a specific symptom, and (2) quotations explicitly referring to specific symptoms, which were then coded accordingly. A codebook for qualitative data was developed in an Excel spreadsheet based on the ninth edition of the Manual for Assessment and Documentation of Psychopathology in Psychiatry (AMDP) system, a well-established manual widely used for the systematic assessment of psychopathology and psychiatric symptoms in clinical practice.^16^ The AMDP was selected as a phenomenological anchor to organize symptom-related accounts for clinical readability, rather than as a rigid classificatory scheme, because it represents one of the few cross-diagnostic, comprehensive frameworks available for characterizing psychiatric symptoms from a patient-centered perspective, making it particularly well-suited for synthesizing diverse lived experiences across SSDs.^17^ Each participant quote was tagged according to the corresponding AMDP symptom domain. In cases where ambiguity persisted in assigning a symptom tag, the descriptions and interpretations provided by the authors of the original study were considered as the primary reference.

To synthesize the results, we applied a three–step thematic synthesis: (1) coding the extracted qualitative data, (2) organizing the codes into patterns to form descriptive themes, and (3) developing analytical themes that incorporate and reflect the researchers’ interpretation.^18^

## Results

A total of 6377 records were retrieved from the databases, of which 94 were assessed for eligibility. Ultimately, 53 reports from 52 studies were included in the final analysis. The main reasons for exclusion were reporting quantitative data only (*n* = 2), different or unreported diagnoses (*n* = 22), data aggregated across diagnoses (*n* = 9), outcomes outside the scope of the review (*n* = 5), ineligible study design (*n* = 2), and population not meeting inclusion criteria (*n* = 1). The screening process is detailed in Figure 1. Among the studies included, 90.4% employed qualitative methodologies (semi-structured interviews, in-depth interviews, or focus groups), whereas 9.6% adopted a mixed-methods design. In total, 2856 individuals with a SSD were included. Further study characteristics are detailed in Appendix SC. Results from the two thematic analyses are reported below. It is important to note that the distinction between “general illness experience” and “symptom experience” reflects an analytic choice rather than a separation inherent to participants’ accounts. This distinction was introduced to allow a symptom-by-symptom thematic analysis of how specific symptoms were experienced. At the same time, many narratives described psychosis more broadly, without clear reference to a single identifiable symptom. We retained these accounts in a separate analysis, as they captured important dimensions of lived experience that were often deeply intertwined with symptom experience.

### Thematic Analysis for General Illness Experience

In several instances, participants described their overall experience of SSDs rather than focusing on a specific symptom. These narratives were deemed valuable for contextualizing perceptions of individual symptoms and were therefore extracted and synthesized separately. The analysis generated a total of 345 codes. The resulting themes and subthemes, together with illustrative quotations, are reported in Table 1. A table with complete explanation for each sub-theme is reported supplementary materials (Appendix SC).

### Thematic Analysis for Symptom Experience

A total of 766 codes were generated from participants’ accounts of their symptom experiences. Each code was assigned to a specific symptom definition based on the AMDP system, which offers a detailed phenomenological framework for their classification. Following the AMDP manual, symptoms were subsequently grouped into dimensions to ensure consistency; however, for readability across clinicians, symptoms are also referred to using broader categories (positive, negative, affective, cognitive, and dissociative) throughout the text. Thematic analysis conducted for each symptom yielded one or more themes, summarized in Table 2. A comprehensive table with detailed explanations of each theme is presented in Appendix SC.

**Table 2:** Thematic Analysis Based on the AMDP System^a^

Dimension	Symptom	Definition	Quote	Themes
Disorders of perceptions	Hearing voices	Perception of voices without someone speaking	“There’s literally a person inside of my head, speaking”^51^	(1) Phenomenological characteristics; (2) Fear, sadness, unpleasant emotions;(3) Pleasant emotions; (4) Coping and meaning making
	Other auditory hallucinations	Perception of nonverbal hallucinatory sounds or noises.	“I could hear like noises, like of a rustle outside the door”^52^	(1) Human, animal or environmental sounds
	Visual hallucinations	Visual perceptions without corresponding external stimuli	“I see a figure in a cloud or something and I see a gun pointed at me”^53^	(1) Threatening and violent behavior; (2) Truth and subjugation; (3) Blurred and dreamy reality
	Bodily hallucinations	Tactile perception without corresponding stimuli, or altered bodily perceptions	“There were worms inside the nose and the nerves were eaten up”^54^	(1) Intrusion; (2) Altered body parts; (3) Painful sensations
	Other hallucinations	N.R.	“It [hallucinatory experience] felt as if it exceeded consciousness”^55^	(1) Different reality; (2) Meaningful experience; (3) Coping strategies; (4) Suicidality, distress, unpleasant emotions
Delusions	Delusion of grandiosity	Delusional exaggerated self-esteem or self-aggrandizement, or the belief of having special powers or skills.	“I am a Greek Princess or Sita.”^54^	(1) Transformed identity and superhuman status; (2) Social consequences and risky situations; (3) Power, agency, protective purposes; (4) Repetitive thinking
	Delusion of guilt	Delusional conviction of having failed in one’s duty, committed a crime or sin, or having wronged others.	“I’m a sinner whom Jesus died and suffered for.”^56^	(1) Religion and sin; (2) Compulsions
	Delusion of persecution	The patient experiences themself as the target of hostilities or monitoring. They have delusions that they are threatened, offended, insulted, mocked, under surveillance or derided by others who are after their money and belongings or out to destroy their health or even their life.	“I feel as though I have passed on information to the relevant intelligence organization through the quiz I do on a Monday evening”^57^	(1) Surveillance and control; (2) Distrust and fear of harm; (3) Overwhelmed and suicidal
	Delusion of reference	The delusional conviction that environmental events or objects are intended to be of special significance to the patient. Delusional attribution of events to one’s self.	“If I be watching TV, I’ve got to turn that off too because I can’t watch it too long because it seems like they’re saying, like the story is me, all about me”^31^	(1) Emotional pain; (2) Social withdrawal and unusual behavior
	Delusional ideas	Persistent or enduring delusional thoughts and convictions	“Now, I can see that it makes no sense that my frontal lobes are made of starlight, but I still have a feeling deep inside, believing that this is the case.”^55^	(1) Different reality
	Delusional mood	Delusional mood indicates an unusual, often confused, and tense emotional state of mind preceding manifestation of the delusion. The mood consists of attributing significance to, and making connections between, unsubstantiated guesses, suppositions, and expectations, which, to the healthy person, have no meaning or relevance.	“There’s a whole different set of everything”^58^	(1) Different reality; (2) Existential revelations and spiritual dimensions; (3) Fear, loss of control, self-harm
	Other delusions	Delusions that do not fit in any of the above categories	“Girls have cancer in their mouth and if I kiss them I will get surely ill”^54^	(1) Religious content; (2) Illness-related and somatic content; (3) Identity transformation; (4) Control and manipulation
Worries and compulsions	Suspiciousness	The behavior of others is viewed with anxiety, mistrust, or hostility and perceived as directed at one’s own person	“I’d be cautious around everything, even the people I’m close to”^59^	(1) Paranoia and fear
Ego boundary disturbances	Thought broadcasting	The patient’s personal thoughts are experienced as no longer belonging to the patient alone but accessible by others who will know what the patient is thinking (mind reading). This can be a passive experience with the patient believing their thoughts as accessible to others, without someone else actively attempting to read their mind.	“It’s fun, isn’t it, that they can hear everything?”^60^	(1) Loss of boundaries
	Depersonalization	The patient perceives themself as alien, unreal, changed, or as a stranger.	“You’re on automatic pilot and you’re an observer”^61^	(1) Emptiness and disconnection
	Derealization	The patient experiences surroundings or time as if they are unreal and changed; all feelings of familiarity with and trust in the environment are lost.	“I thought it was all a film and that I was actually only a small pawn within that film”^61^	(1) Feeling stuck in a dream; (2) Estrangement and disconnection
Formal thought disorders	Pressured thinking	The patient feels helplessly exposed to the pressures of floods of different ideas or thoughts.	“I start crying…because the thoughts are so scary and bad and you don’t know what’s going on in your head”^31^	(1) Fear
	Inhibited thinking	The thinking process is subjectively experienced by the patient as being slowed down or blocked (as by an inner wall of resistance).	“There is absolutely nothing in your head and, well yeah, you’re not thinking”^48^	(1) Emptiness
	Rumination	Endless mental preoccupation with, or excessive concern over, mostly unpleasant thoughts.	“...I’m just thinking about it constantly ...I can’t put my mind onto things I want to think about, I just think about that all the time.”^62^	(1) Psychological and behavioral distress; (2) Intrusiveness
	Flight of ideas	An increasing multitude of thoughts and ideas which, however, are no longer firmly guided by clear goal-directed thinking. As a result of diverse associations, thinking goals change frequently or become lost.	“Thoughts went in all directions”^45^	(1) Inability to control thoughts
	Incoherence/Derailment	The interviewer is unable to establish sensible connections between the patient’s thinking and verbal output, which is sometimes also called derailment. In extreme cases, what remains is a seemingly arbitrary tumble of fragmented sentences, phrases, and thoughts.	“I started answering like one and one equals two. I was going like this making arrows and pluses.”^45^	(1) Disconnected, illogical
	Thought blocking	Sudden disruption of an otherwise normal flow of thought or speech for no obvious reason.	“It’s like confusion. I can’t say what I want to say because my head goes blank.”^31^	(1) Confusion
Disturbances of affect	Loss of feelings	The patient reports a reduction in or loss of affective experience and a subjectively felt emotional emptiness. The patient experiences themself as deprived of feelings, inwardly empty and numb, both in terms of positive feelings such as joy and happiness, but also negative emotional states such as sadness and despair.	“I didn’t feel like detached. And I, I didn’t feel like anything”^63^	(1) Numbness
	Blunted affect	The number (spectrum) of affective states displayed is diminished	“Facial expressions? I haven’t got many expressions...”^64^	(1) Reduced emotional expression; (2) Withdrawal
	Depressed mood	Negatively tinged affective state characterized by low mood, sadness and dejection	“I was very embarrassed, humiliated, disgusted, and depressed over my illness”^31^	(1) Burden and negative self-perception; (2) Illness-related depression
	Irritability	The patient reacts in an inappropriately swift or fierce manner with anger and/or aggression	“I get very angry in certain situations. I’m easy to upset.”^31^	(1) Hurtful sensitivity
	Affective incontinence	Affective reactions/expressions may become overbearing at the slightest impulse, remain beyond the control of the patient and at times assume an exaggerated level of intensity (lack of affective self-control).	“I feel sad, sensitive. I break just like glass if you say anything bad to me.”^31^	(1) Feeling vulnerable; (2) Anxiety and fear
Disorders of consciousness	Expanded consciousness	This is a qualitative dimension of consciousness disturbances, where patients report instances of heightened or intensified awareness of inner and outer events.	“A sound that normally seems to be in the background, becomes a sound that cuts you and which enters sharply”^48^	(1) Intensified perceptions; (2) Mystical experiences
Additional psychopathological items	Hearing one’s own thoughts	Hearing one’s own thoughts in clearly auditory, fully verbalized form	“Basically what it is, is my thoughts, in the form of a voice.”^31^	(1) Inner voice
	Acceleration of thinking	Subjective perception of experiencing accelerated thought processes	“My thoughts were so numerous that I didn’t manage talking to people.”^45^	(1) Social impairment; (2) Cognitive overload
Disorders of drive	Inhibition of drive	The patient experiences levels of energy and personal initiative as stalled or blocked	“I can’t function. I’ll shut right down”^31^	(1) Feeling shut down
	Lack of drive	Lack of energy, initiative and interest	“I wasn’t moving, I was sitting down… I wasn’t talking. I was just like, you know, like a zombie”^63^	(1) Zombie-like state; (2) External hindrance
	Mannerism	Everyday movements and actions (also physical gestures, facial expressions and speech) appear to the outside observer as eccentric, cranky, showy and forced; they are sometimes performed in a distinctive/exaggeratedly playful manner.	“I was half bent over, and walked in this way. I couldn’t control it and people commented about it. They said I must be ill or fucked up. They paid attention to me because I behaved weird”^65^	(1) Feeling judged
Other disturbances	Social withdrawal	Withdrawal from other people’s company	“[Patient’s name] reported that he was withdrawing because his ‘environment had difficulties with me, to see me like a full person.’”^48^	(1) Seeking solitude; (2) Fear of stigma
	Self-harm	Self-harming without suicidal intent.	“I cut my face ‘cos I wanted to look different, I cut my face with a razor”^44^	(1) Non-acceptance
	Aggressiveness	Hostile, offensive and attacking behavior.	“I was so mad, I just had to hit it.”^66^	(1) Environmental and relational triggers; (2) Self-regulation attempts; (3) Frustration and powerlessness
Disturbances of attention and memory	Memory impairment	Reduction in or loss of ability to store information in the memory for longer than 10 minutes or recall previously studied material from memory.	“I do, do crosswords with my mum every Sunday afternoon”^67^	(1) Trying coping strategies
	Disturbed concentration	Reduced ability to keep one’s attention and remain focused on an activity or topic for a prolonged period of time.	“Concentration ...dreadful ...absolutely dreadful. Couldn’t watch TV program; couldn’t listen to the radio couldn’t even listen to music.”^64^	(1) Impairment in daily living

^a^A complete table with example quotes for each theme is reported in Appendix SC (Table C.2).

#### Disorders of Perceptions

This section presents hallucinations occurring in any sensory modality, described by participants in a wide range of diverse ways. Hearing voices emerged as a highly heterogeneous experience, yielding multiple themes. Some participants described insulting and threatening voices associated with fear and distress, whereas others experienced them as comforting companions offering guidance. Several attempted to make sense of the voices by linking them to aspects of their own personality. Many also perceived the voices as distinct entities, each with its own recognizable traits and characteristics. Other reported auditory hallucinations included indistinct human voices, animal sounds, and noises resembling those from the external environment. Visual hallucinations were described from some participants as dream-like, while others perceived them as entirely realistic; in general, they were frequently accompanied by intense feelings of terror. Bodily hallucinations were reported as sensations of having foreign entities, often worms or insects, inside the body, perceiving internal organs as moving, or experiencing vivid sensations of physical pain. In non-specific terms, hallucinatory experiences were described by some participants as highly distressing and, in certain cases, associated with suicidal ideation, whereas others characterized them as enriching and personally meaningful.

#### Delusions

Among delusional experiences, only grandiose delusions were occasionally perceived as positive, being associated with feelings of power, superiority, and elevated mood. More broadly, delusions were described as living in an altered reality, often accompanied by distressing emotions such as fear, loss of control, and overwhelming sensations.

#### Ego Boundary Disturbances

This dimension includes phenomena that involve alterations in the experience of the self in relation to internal thoughts or external reality.^19^ They reflect phenomenologically distinct experiences that may involve both alterations of the minimal self, that is, the immediate sense of ownership and agency, and disturbances of the narrative self, relating to the coherence of identity over time.^20^^,^^21^ However, this grouping finds rationale in the emerging themes. Thought broadcasting, which might be considered a positive symptom, is described by participants as the experience of loss of self-boundaries, whereby thoughts are experienced as no longer private or fully self-contained. By contrast, depersonalization and derealization were more frequently described as experiences of estrangement from oneself or the external world, often conveyed through metaphors such as feeling as if in a dream, on “automatic pilot” or in a “zombie-like” state. These experiences may reflect broader disruptions in identity, continuity of experience, and reality perception. Across these accounts, disconnection from oneself and the external world emerged as a central feature.

#### Formal Thought Disorders

This dimension comprised a range of disturbances affecting the flow, organization, and controllability of thoughts. Participants described pressured thinking, which was commonly associated with a sense of fear, as thoughts became rapid and overwhelming. In contrast, inhibited thinking was often experienced as emptiness, characterized by an inability to generate or sustain thoughts. Rumination emerged as a prominent feature, described as both intrusive and associated with significant psychological and behavioral distress, as individuals felt trapped in repetitive, distressing thought cycles. Additionally, flight of ideas was frequently linked to an inability to control thoughts, with participants reporting that their thinking shifted quickly and unpredictably. Experiences of incoherence or derailment reflected disconnected and illogical patterns of thought, which often impaired communication and clarity. Finally, thought blocking was described in relation to confusion, as individuals experienced sudden interruptions in their thinking, disrupting both internal thought processes and conversational flow.

#### Disorders of Affect

This dimension refers to disturbances in mood and emotional expression, frequently reported in schizophrenia and closely intertwined with both positive and negative symptoms.^22^ From our data, depressed mood, irritability and affective incontinence were reported. Depressive symptoms were described both as an independent manifestation of psychopathology and as a consequence of illness insight. In the first case, participants reported low mood emerging after the resolution of a psychotic episode. In the second, depressive feelings were linked to increased awareness of the disorder and the accompanying fear of being perceived as different or incapable of leading a normal life. Irritability was reported as extreme sensitivity in certain situations, and affective incontinence was linked to anxiety and fear.

#### Other Psychopathological Dimensions

Additional psychopathological dimensions also emerged, including alterations in consciousness, suspiciousness, accelerated thinking, hearing one’s own thoughts, disturbances of drive, withdrawal, aggression, and cognitive difficulties. Disturbances of drive were experienced as a feeling of being “shut down” or existing in a “zombie-like” state. Overall, participants described disorders of drive as a pervasive sense of numbness and emptiness affecting both thoughts and emotions, which is perceived as a different experience compared to dissociative symptoms. Suspiciousness was also experienced with fear. Hearing one’s own thoughts was described as the peculiar experience of perceiving the inner voice audibly yet distinguished from verbal hallucinations. Expanded consciousness was reported either as an intensification of sensory perceptions or as the experience of a mystical state. Social withdrawal was reported not only as a personal need to be alone but also because of illness awareness and the accompanying fear of stigma; withdrawal was also an emerging theme from blunted affect. Accelerated thinking was commonly described as an inability to structure thoughts, which in turn hindered communication with others. Among cognitive symptoms, memory and attention were reported as impaired in our results. On the one hand, participants described trying to cope by engaging in small cognitive exercises, such as crossword puzzles; on the other, they emphasized their inability to carry out ordinary recreational activities, including reading a book or watching a film. No specific references were found regarding executive functioning impairment. Another dimension that emerged was aggression, expressed both as self-directed and other-directed behavior. When self-harm was explicitly described as a primary symptom, rather than because of positive symptoms, it was associated with a negative self-perception. Regarding aggressive behavior, some subjects identified external triggers that intensified anger, such as the actions of others or the experience of being confined in hospital. These situations were closely linked to feelings of frustration and powerlessness over their condition, which in turn fueled aggressiveness. Conversely, other subjects reported engaging in self-regulation strategies when anger arose, to prevent aggressive outbursts.

#### Quality Assessment

All included studies demonstrated methodological robustness, with CASP scores ranging from 6 to 10. The main concern emerged with item 6, concerning the relationship between the researcher and participants, which was not explicitly reported in the 79.2% of studies. Detailed tables with CASP items scores are reported in the supplementary materials (Appendix SD).

## Discussion

A wide range of themes emerged from participants’ interviews on their lived experience of schizophrenia. The theme of disorder impact (1) underscored how schizophrenia permeates physical, psychological, social, and relational life and connects closely with isolation and stigma (2). Prior studies similarly highlight stigma as pervasive, with some subjects even preferring depression to psychosis.^23^^,^^24^ Another theme (3) concerned relationships with pharmacological treatment, clinicians, and the healthcare system, with reports of poor communication and coercive practices during hospitalization, including coercive intramuscular antipsychotic injections.^25^ This aligns with recent qualitative reviews of lived experiences among people with schizophrenia receiving oral and long–acting injectable antipsychotics, which highlight how service-related factors strongly influence treatment experiences and ongoing engagement.^26^^,^^27^ Stigma among healthcare professionals, manifested through stereotypes, prejudices, and discriminatory behaviors, may contribute to these negative perceptions.^28^ Theme (4) captured despair and the experience of disruption and fragmentation of self, external reality, and meaning. Theme (5) concerned coping in daily life and the protective role of social networks; consistent with prior work, social support enhances coping and resilience, whereas its absence predicts poorer outcomes.^29^ The symptom–level thematic synthesis identified overarching patterns across domains. Fear recurred throughout participants’ accounts (auditory hallucinations, persecutory ideas, suspiciousness, pressured thinking, social withdrawal, and affective incontinence) indicating its cross-cutting relevance to positive, negative, emotional, and social functioning. Within positive symptoms, many described inhabiting an altered reality spanning hallucinations, delusional ideas, and delusional mood. In the cognitive domain, the absence of explicit references to executive deficits may seem unexpected given their centrality in cognitive dysfunction in schizophrenia.^30^ This might be related to the fact that participants often linked difficulties in organizing and planning daily activities to negative or affective symptoms, suggesting that when executive dysfunction is present, it may be experienced and reported primarily through emotional and motivational lenses.^31^ Within the AMDP category “other delusions,” four recurrent patterns (somatic, identity transformation, control, and religious non–grandiose delusional content) may warrant recognition as autonomous categories to better reflect subjects’ experiences. Convergence with prior syntheses further supports the present themes: qualitative work on negative symptoms highlights isolation, social withdrawal, and stigma, and reviews of delusions emphasize grounding their understanding in lived experience beyond categorical labels.^32^^,^^33^ Most reported data described positive symptoms such as hallucinations and delusions, reflecting the focus of many included studies, and thus indicating the need for qualitative research to explore the other, under-investigated dimensions of schizophrenia. Importantly, these findings should not be understood as an attempt to revise existing diagnostic categories, but rather as offering a patient-centered perspective that extends beyond them. In this context, the AMDP framework was used as a descriptive and phenomenologically oriented structure to organize symptom-related accounts at the level of individual symptoms, rather than as a diagnostic model. The synthesis showed that several experiential dimensions (particularly fear, altered reality, and disconnection) extended across symptom categories, suggesting that lived experience does not always conform to discrete diagnostic boundaries. Moreover, some experiential dimensions may resist stable categorization altogether. This highlights the limits of classification itself and suggests that lived experience should not only be organized into symptom categories but also approached as a complex and sometimes irreducible source of clinical knowledge. Beyond diagnostic considerations, these findings have important implications for clinical practice. The pervasive role of fear across symptom domains suggests that clinical assessment should move beyond the identification of symptoms to more systematically explore the subjective meaning and emotional impact of psychotic experiences. Similarly, the ambivalence participants expressed toward medication and services highlights the need to interpret disengagement not simply as non-adherence, but as a meaningful response shaped by prior experiences, perceived coercion, and concerns about identity and autonomy. These findings underscore the importance of therapeutic approaches grounded in dialog, shared understanding, and collaborative decision-making, as well as the development of more person-centered models of care. In this context, psychological and psychosocial interventions may play a key role: cognitive behavioral therapy for psychosis may help address threat-related appraisals and distress,^34^^,^^35^ metacognitive approaches may support meaning-making and the reconstruction of a coherent sense of self,^36^^,^^37^ and psychosocial interventions targeting social functioning may help address the relational and social dimensions of the disorder.^38^^,^^39^ These patient–reported qualitative findings highlighted symptoms not traditionally viewed as core, yet experienced as highly relevant, underscoring the importance of moving beyond diagnostic criteria to develop patient-tailored assessments and more comprehensively evaluate responses to pharmacological treatment.

### Limitations and Strengths

Our work has some limitations that should be acknowledged. First, as with all qualitative syntheses, our findings are shaped by the quality and heterogeneity of the included studies, which varied in methodology, sample characteristics, and reporting detail. In addition, the included studies spanned different geographical regions, service contexts, illness stages, and participant characteristics, which may influence how psychosis, stigma, and treatment are experienced and reported. While the synthesis aim ed to identify common experiential patterns, this contextual heterogeneity should be considered when interpreting the generalizability of the findings. Second, most data were derived from interview excerpts, which may be influenced by recall bias, social desirability, and the interpretative stance of the researchers. Moreover, CASP appraisal showed frequent under-reporting of the researcher–participant relationship; future studies should explicitly describe and reflect on this aspect. The present findings could also be extended through co-produced or lived experience–led qualitative syntheses, particularly to explore dimensions of meaning-making, identity, and relationality that may not map neatly onto existing psychopathological frameworks. Despite these limitations, the study also has notable strengths. To our knowledge, this is the first qualitative meta-synthesis to systematically integrate subjects’ lived experiences with both the illness and its core symptoms. The use of a rigorous thematic synthesis methodology, together with cross-validation against existing diagnostic systems, enhances the robustness and clinical relevance of our conclusions.

## Conclusion

This meta-synthesis highlights the complexity of SSDs as experienced and described by subjects themselves. Beyond the traditional dichotomy of positive and negative symptoms, individuals described a wide range of affective, cognitive, dissociative, and behavioral experiences that align with the broader dimensional framework proposed in ICD-11 and DSM-5. These experiences profoundly shape daily life and impair functional autonomy. Themes such as fear, stigma, fragmentation of self and reality, and the pivotal role of social support and therapeutic relationships emphasize the need for diagnostic systems and clinical care to better integrate lived experience. By grounding our understanding of SSDs in subjects’ perspectives, future research and practice may move toward more comprehensive, patient-centered frameworks that improve both the accuracy of diagnosis and the quality of treatment and care.

## Supplementary Material

supplementary_symptoms_bulletin_sbag122
