# Supplementary material for: Reconceptualizing Schizophrenia through a Patient-Oriented Perspective: a Systematic Review and Qualitative Meta-Synthesis
Source: Schizophr Bull. 2026 Sep 25;52(5):sbag122. doi: 10.1093/schbul/sbag122 (PMC13612232; doi:10.1093/schbul/sbag122)
Supplement: Supplementary file 1 [file supplementary_symptoms_bulletin_sbag122.docx]

**Supplementary materials**

**Appendix A: search strategy**

Search strategy (PubMed): #1 AND #2 AND #3

#1 "schizophrenia"[MeSH Terms] OR "paranoid disorders"[MeSH Terms] OR "schizo*"[Title/Abstract] OR "psychot*"[Title/Abstract] OR "psychos*"[Title/Abstract] OR "thought disorder*"[Title/Abstract] OR "delusion*"[Title/Abstract] OR "hallucination*"[Title/Abstract] OR "positive symptom*"[Title/Abstract] OR "negative symptom*"[Title/Abstract] OR "antipsychotic*"[Title/Abstract] OR "neuroleptic*"[Title/Abstract] OR "disorganized symptom*"[Title/Abstract] OR "disorganization syndrome"[Title/Abstract] OR "cognitive disorganization"[Title/Abstract] OR "disorganized thinking"[Title/Abstract]

#2 ("negative symptom*"[Title/Abstract] OR "negative syndrome*"[Title/Abstract] OR "deficit syndrome*"[Title/Abstract] OR "affective flat*"[Title/Abstract] OR "flat* affect"[Title/Abstract] OR "affective blunt*"[Title/Abstract] OR "blunt* affect"[Title/Abstract] OR "alogia"[Title/Abstract] OR "speech poverty"[Title/Abstract] OR "poverty of speech"[Title/Abstract] OR "anhedon*"[Title/Abstract] OR "amotivation"[Title/Abstract] OR "motivation difficult*"[Title/Abstract] OR "poor motivation"[Title/Abstract] OR "avolition"[Title/Abstract] OR "apath*"[Title/Abstract] OR "asociality"[Title/Abstract] OR "positive symptom*"[Title/Abstract] OR "hallucination*"[Title/Abstract] OR "delusion*"[Title/Abstract] OR "paranoia"[Title/Abstract] OR "thought disorder"[Title/Abstract] OR "disorganized speech"[Title/Abstract] OR "disorganized behavior"[Title/Abstract] OR "incoherence"[Title/Abstract] OR "derailment"[Title/Abstract] OR "tangentiality"[Title/Abstract] OR "neologism*"[Title/Abstract] OR "cognitive symptom*"[Title/Abstract] OR "cognitive deficit*"[Title/Abstract] OR "working memory"[Title/Abstract] OR "attention deficit*"[Title/Abstract] OR "executive function*"[Title/Abstract])

#3 ("Document Analysis"[Title/Abstract] OR "focus groups"[Title/Abstract] OR "interviews as topic"[Title/Abstract] OR "narration"[Title/Abstract] OR "qualitative research"[Title/Abstract] OR ethnograph*[Title/Abstract] OR "field work"[Title/Abstract] OR fieldwork[Title/Abstract] OR "focus group"[Title/Abstract] OR "focus groups"[Title/Abstract] OR "key informant"[Title/Abstract] OR "key informants"[Title/Abstract] OR "qualitative research"[Title/Abstract] OR "qualitative studies"[Title/Abstract] OR "qualitative study"[Title/Abstract] OR "qualitative synthesis"[Title/Abstract] OR "depth discussion"[Title/Abstract] OR "depth discussions"[Title/Abstract] OR "depth interview"[Title/Abstract] OR "depth interviews"[Title/Abstract] OR "depth questionnaire"[Title/Abstract] OR "depth questionnaires"[Title/Abstract] OR "face discussion"[Title/Abstract] OR "face discussions"[Title/Abstract] OR "face interview"[Title/Abstract] OR "face interviews"[Title/Abstract] OR "face questionnaire"[Title/Abstract] OR "face questionnaires"[Title/Abstract] OR "group discussion"[Title/Abstract] OR "group discussions"[Title/Abstract] OR "group interview"[Title/Abstract] OR "group interviewed"[Title/Abstract] OR "group interviews"[Title/Abstract] OR "group questionnaire"[Title/Abstract] OR "group questionnaires"[Title/Abstract] OR "groups interviewed"[Title/Abstract] OR "guided discussion"[Title/Abstract] OR "guided discussions"[Title/Abstract] OR "guided interview"[Title/Abstract] OR "guided interviews"[Title/Abstract] OR "guided questionnaire"[Title/Abstract] OR "guided questionnaires"[Title/Abstract] OR "indepth discussion"[Title/Abstract] OR "indepth discussions"[Title/Abstract] OR "indepth interview"[Title/Abstract] OR "indepth interviews"[Title/Abstract] OR "indepth questionnaire"[Title/Abstract] OR "indepth questionnaires"[Title/Abstract] OR "semistructured discussion"[Title/Abstract] OR "semistructured discussions"[Title/Abstract] OR "semistructured interview"[Title/Abstract] OR "semistructured interviews"[Title/Abstract] OR "semistructured questionnaire"[Title/Abstract] OR "semistructured questionnaires"[Title/Abstract] OR "structured discussion"[Title/Abstract] OR "structured discussions"[Title/Abstract] OR "structured interview"[Title/Abstract] OR "structured interviews"[Title/Abstract] OR "structured questionnaire"[Title/Abstract] OR "structured questionnaires"[Title/Abstract] OR "unstructured discussion"[Title/Abstract] OR "unstructured discussions"[Title/Abstract] OR "unstructured interview"[Title/Abstract] OR "unstructured interviews"[Title/Abstract] OR "unstructured questionnaire"[Title/Abstract] OR "unstructured questionnaires"[Title/Abstract] OR "Patient Experience"[Title/Abstract] OR "Patient Experiences"[Title/Abstract] OR "Patient Belief"[Title/Abstract] OR "Patient Beliefs"[Title/Abstract] OR "Patient Attitude"[Title/Abstract] OR "Patient Attitudes"[Title/Abstract] OR "Patient Perspective"[Title/Abstract] OR "Patient Perspectives"[Title/Abstract] OR "Patients Experience"[Title/Abstract] OR "Patients Experiences"[Title/Abstract] OR "Patients Belief"[Title/Abstract] OR "Patients Beliefs"[Title/Abstract] OR "Patients Attitude"[Title/Abstract] OR "Patients Attitudes"[Title/Abstract] OR "Patients Perspective"[Title/Abstract] OR "Patients Perspectives"[Title/Abstract] OR "Service User Experience"[Title/Abstract] OR "Service User Experiences"[Title/Abstract] OR "Service User Belief"[Title/Abstract] OR "Service User Beliefs"[Title/Abstract] OR "Service User Attitude"[Title/Abstract] OR "Service User Attitudes"[Title/Abstract] OR "Service User Perspective"[Title/Abstract] OR "Service User Perspectives"[Title/Abstract] OR "Service Users Experience"[Title/Abstract] OR "Service Users Experiences"[Title/Abstract] OR "Service Users Belief"[Title/Abstract] OR "Service Users Beliefs"[Title/Abstract] OR "Service Users Attitude"[Title/Abstract] OR "Service Users Attitudes"[Title/Abstract] OR "Service Users Perspective"[Title/Abstract] OR "Service Users Perspectives"[Title/Abstract] OR "Patient Preference"[Title/Abstract] OR "Patient Preferences"[Title/Abstract] OR "Patients Preference"[Title/Abstract] OR "Patients Preferences"[Title/Abstract] OR "Service User Preference"[Title/Abstract] OR "Service User Preferences"[Title/Abstract] OR "Service Users Preference"[Title/Abstract] OR "Service Users Preferences"[Title/Abstract] OR "Thematic analys*"[Title/Abstract] OR "Patient Opinion"[Title/Abstract] OR "Patient Opinions"[Title/Abstract] OR "Patients Opinion"[Title/Abstract] OR "Patients Opinions"[Title/Abstract] OR "Service User Opinion"[Title/Abstract] OR "Service User Opinions"[Title/Abstract] OR "Service Users Opinion"[Title/Abstract] OR "Service Users Opinions"[Title/Abstract] OR "Patient Evaluation"[Title/Abstract] OR "Patient Evaluations"[Title/Abstract] OR "Patients Evaluation"[Title/Abstract] OR "Patients Evaluations"[Title/Abstract] OR "Service User Evaluation"[Title/Abstract] OR "Service User Evaluations"[Title/Abstract] OR "Service Users Evaluation"[Title/Abstract] OR "Service Users Evaluations"[Title/Abstract] OR "Thematic synthes*"[Title/Abstract] OR "Grounded theor*"[Title/Abstract] OR "Subjective experience"[Title/Abstract] OR "Subjective Experiences"[Title/Abstract])

**Appendix B: exclusion reasons**

**Table B.1** Reasons for excluded studies

| **Title** | **Year** | **Authors** | **Exclusion reason** |
| --- | --- | --- | --- |
| Patient recall of intensive care delirium: A qualitative investigation | 2024 | la Cour, K.N. and Andersen-Ranberg, N.C. and Mortensen, C. and Poulsen, L.M. and Mathiesen, O. and Egerod, I. and Collet, M. | Wrong or missing diagnosis |
| Narrative Forewarnings: A Qualitative Analysis of the Themes Preceding Disorganized Speech in Schizophrenia | 2024 | Bettis, R.J. and Faith, L.A. and Beard, A.M. and Whan, B.A. and Hegwood, C.M. and Monette, M.A. and Myers, E.J. and Linton, I.S. and Leonhardt, B.L. and Salyers, M.P. and Minor, K.S. | Wrong outcome |
| Visual hallucinations in psychosis: What do people actually see? | 2025 | Aynsworth, C. and Waite, F. and Sargeant, S. and Humpston, C. and Dudley, R. | Wrong or missing diagnosis |
| Making Sense of Spiritual, Metaphysical, and Eschatological Elements in Delusions: A Qualitative Study Using Interpretative Phenomenological Analysis | 2024 | Nischk, D. and Gutschmidt, R. | Wrong or missing diagnosis |
| “Distress is probably the wrong word”: exploring uncertainty and ambivalence in non-clinical voice-hearing and the psychosis continuum | 2024 | Swyer, A. and Woods, A. and Ellison, A. and Alderson-Day, B. | Wrong or missing diagnosis |
| Voice hearers' explanations of trauma-related voices and processes of change throughout imagery rescripting: A qualitative exploration | 2023 | Strachan, L.P. and Paulik, G. and Roberts, L. and McEvoy, P.M. | Data aggregated across diagnosed |
| Features of Representations and Subjective Experience of the Images of Hallucinations, Fantasies, Dreams, and Imagination in Patients with Mental Disorders | 2023 | Shilko, N.S. and Omelchenko, M.A. and Ivanova, E.M. and Enikolopov, S.N. | Data aggregated across diagnosed |
| In the eyes of the beholders: Subjective experiences of positive symptoms among patients with symptoms of psychosis seeking psychotherapy | 2023 | Schneider, B.C. and Rahmede, M. and Pillny, M. and Karow, A. and Moritz, S. and Veckenstedt, R. | Quantitative |
| Characteristics of non-clinical hallucinations: A mixed-methods analysis of auditory, visual, tactile and olfactory hallucinations in a primary voice-hearing cohort | 2020 | Toh, W.L. and Thomas, N. and Robertson, M. and Rossell, S.L. | Wrong or missing diagnosis |
| Psychotic Experiences and Alternate Dimensions: A Thematic Analysis Exploring Frameworks of Psychotic Symptoms Among Saudis | 2021 | Hyder, S. and Almutlaq, N. and Naseem, M.T. and Bilal, L. and Al-Subaie, A. and Al-Habeeb, A. and Altwaijri, Y. | Wrong or missing diagnosis |
| Subjective experiences of cognitive functioning in early psychosis: a qualitative study | 2019 | Wright, A.L. and Phillips, L.J. and Bryce, S. and Morey-Nase, C. and Allott, K. | Data aggregated across diagnosed |
| Experiencing bird voices as auditory hallucinations - Phenomenological lessons from Phylogeny & Ethnography | 2019 | Sadh, K. and Mehta, U.M. and Muralidharan, K. and Shivashankar, N. and Jain, S. | Data aggregated across diagnosed |
| Negative symptoms and therapeutic connection: A qualitative analysis in a single case study with a patient with first episode psychosis | 2018 | Belanger, E.A. and Leonhardt, B.L. and George, S.E. and Firmin, R.L. and Lysaker, P.H. | Wrong outcome |
| “Did I push myself over the edge?”: Complications of agency in psychosis onset and development | 2016 | Jones, N. and Shattell, M. and Kelly, T. and Brown, R. and Robinson, L. and Renfro, R. and Harris, B. and Luhrmann, T.M. | Data aggregated across diagnosed |
| The experience of voice hearing and the role of self-help group: An interpretative phenomenological analysis | 2017 | Rácz, J. and Kaló, Z. and Kassai, S. and Kiss, M. and Pintér, J.N. | Wrong or missing diagnosis |
| Experiences of continued presence: On the practical consequences of 'hallucinations' in bereavement | 2016 | Hayes, J. and Leudar, I. | Wrong or missing diagnosis |
| Experiences of hearing voices: Analysis of a novel phenomenological survey | 2015 | Woods, A. and Jones, N. and Alderson-Day, B. and Callard, F. and Fernyhough, C. | Data aggregated across diagnosed |
| Hearing the unheard: An interdisciplinary, mixed methodology study of women's experiences of hearing voices (auditory verbal hallucinations) | 2015 | McCarthy-Jones, S. and Romero, M.C. and McCarthy-Jones, R. and Dillon, J. and Cooper-Rompato, C. and Kieran, K. and Kaufman, M. and Blackman, L. | Wrong or missing diagnosis |
| Existential feelings and subjective experiences in psychosis: A phenomenological description of the sense of reality | 2015 | Troubé, S. | Wrong study design |
| More real than reality': A study of voice hearing | 2008 | Karlsson, L.-B. | Wrong or missing diagnosis |
| Deconstructing delusions: A qualitative study examining the relationship between religious beliefs and religious delusions | 2006 | Drinnan, A. and Lavender, T. | Wrong or missing diagnosis |
| A qualitative research study of the evolution of symptoms in individuals identified as prodromal to psychosis | 2003 | Corcoran, C. and Davidson, L. and Sills-Shahar, R. and Nickou, C. and Malaspina, D. and Miller, T. and McGlashan, T. | Wrong population |
| An experiential perspective on persecutory paranoia: A grounded theory construction | 2007 | Boyd, T. and Gumley, A. | Wrong or missing diagnosis |
| Subjective experiences of persons with schizoaffective disorders | 2006 | Yip, K.-S. | Quantitative |
| Correspondence between delusions and personal goals: A qualitative analysis | 2000 | Rhodes, J.E. and Jakes, S. | Wrong or missing diagnosis |
| ‘It was like a lightning bolt hitting my world’: Feeling shattered in a first crisis in psychosis. | 2022 | Bögle, Sarah and Boden, Zoë | Wrong or missing diagnosis |
| I divide life into different dimensions, one mental and one physical, to be able to handle life, you know?' Subjective accounts of the content of psychotic symptoms. | 2015 | Strand J and Olin E and Tidefors I | Wrong or missing diagnosis |
| Delusion formation as an inevitable consequence of a radical alteration in lived experience | 2020 | Gunn, Rachel and Larkin, Michael | Wrong or missing diagnosis |
| Emotional earthquakes in the landscape of psychosis: An interpretative phenomenology. | 2016 | Hutchins, Jonathan and Rhodes, John and Keville, Saskia | Wrong or missing diagnosis |
| “It’s just a bit like a rollercoaster”: A longitudinal qualitative study exploring a model of the phases of voice hearing. | 2019 | Bogen-Johnston, Leanne and deVisser, Richard and Strauss, Clara and Hayward, Mark | Wrong or missing diagnosis |
| Service user and carer experiences of seeking help for a first episode of psychosis: a UK qualitative study. | 2011 | Tanskanen S and Morant N and Hinton M and Lloyd-Evans B and Crosby M and Killaspy H and Raine R and Pilling S and Johnson S | Wrong or missing diagnosis |
| Self-care symptom management strategies for auditory hallucinations among patients with schizophrenia in Taiwan. | 2006 | Tsai YF and Chen CY | Wrong outcome |
| What is life after psychosis like? Stories of three individuals diagnosed with schizophrenia. | 2021 | Lee, Ying Ying and Liu, Valerie and Verma, Swapna | Wrong study design |
| "Seeing voices": fused visual/auditory verbal hallucinations reported by three persons with schizophrenia-spectrum disorder. | 2006 | Hoffman, R. E. and Varanko, M. | Wrong study design |
| Auditory hallucinations: a comparison between patients and nonpatients. | 1998 | Honig, A. and Romme, M. A. and Ensink, B. J. and Escher, S. D. and Pennings, M. H. and deVries, M. W. | Quantitative |
| "Sometimes I walk and walk, hoping to get some peace." Dealing with hearing voices and sounds nobody else hears. | 2014 | Kalhovde, Anne Martha and Elstad, Ingunn and Talseth, Anne-Grethe | Data aggregated across diagnosed |
| Voice-Hearing Across The Continuum: A Phenomenology of Spiritual Voices. | 2022 | Moseley, Peter and Powell, Adam and Woods, Angela and Fernyhough, Charles and Alderson-Day, Ben | Wrong or missing diagnosis |
| [The experience of auditory hallucination and the role of self-help groups: an interpretative phenomenological analysis]. | 2016 | Kassai, Szilvia and Kiss, Márta and Pintér, Judit Nóra and Rácz, József | Wrong or missing diagnosis |
| From Experience to Symptoms: A Multilayer Hierarchy of Psychopathological Dimensions in Schizophrenia. | 2025 | Lechner S and Sandsten KE and Hirjak D and Daub J and Fritze S and Brandt GA and Arantes-Gonçalves F and Wolman A and Stefanelli R and Gojer J and Gulati S and Hersi H and Parnas J and Northoff G | Wrong outcome |
| The dynamic interplay between anxiety-related, psychotic, and suicidal experiences: a qualitative study. | 2025 | Gooding P and Harris K and Peters S and Haddock G | Wrong outcome |
| "We Are Humans, and We Are People" - A Thematic Analysis Exploring the Disclosure and Help-Seeking Experiences of Young People Who Experience Voice-Hearing Within Mental Health Services in the UK. | 2025 | Ellis M and Hayward M and Berry C and Fowler D and Rammou A | Wrong or missing diagnosis |

**Appendix C: included studies and emerging themes emerging themes**

**Table C.1** Characteristics of included studies

| **Authors** | **Country** | **Aim** | **Design** | **Recruitment setting** | **Diagnosis** | **Diagnosis criteria** | **Sample size (** **SSDs spectrum)** | **Data analysis (qualitative data)** | **Age mean (SD)** | **CASP** |
| --- | --- | --- | --- | --- | --- | --- | --- | --- | --- | --- |
| Alderson-Day et al., 2021 | UK | To investigate personification in individuals' early voice-hearing experiences. | Mixed-methods (semi-structured interviews and PSYRATS) | Early Intervention in Psychosis services | SSDs | NR | 13 | Thematic analysis | 28.70 (9.96) | 9 |
| Boydell et al., 2003 | Canada | To explore motivation in schizophrenia according to a personal point of view | Qualitative (in-depth interviews) | Self-help organization, hospital clinic and place of employment | Schizophrenia | NR | 6 | Hermeneutical analysis | NR | 9 |
| Butcher et al., 2020 | UK | To explore negative symptoms in schizophrenia | Qualitative (semi-structured interviews) | Community and inpatient National Health Service | SSDs | DSM 5, DSM-IV | 20 | Thematic analysis | 52 | 10 |
| Campbell & Morrison, 2007 | UK | To compare schizophrenia subjects experience of paranoia with non-patients experience | Mixed-methods (semi-structured interviews and PDI) | Community mental health service | SSDs | NR | 6 | Interpretative phenomenological analysis (IPA) | 30.9 (12) | 9 |
| Cassaniti et al., 2025 | Thailand | To explore cultural influence in the content of voices | Qualitative (interviews) | Inpatient unit | schizophrenia | NR | 20 | Grounded theory | NR | 9 |
| Chernomas et al., 2000 | Canada | To understand women's perspectives of schizophrenia | Qualitative (focus groups) | Community-based organization | schizophrenia, schizoaffective disorder | NR | 28 | Thematic analysis | NR | 7 |
| Clements et al., 2020 | Australia | To explore lived experience of voice hearing in schizophrenia | Qualitative (semi-structured interviews) | Community mental health  centre. | SSDs | NR | 5 | Thematic analysis | 45.4 (12.72) | 10 |
| Corin, 1998 | Canada | To clarify the challenges of psychiatric deinstitutionalization and identify factors that support community living for individuals diagnosed with schizophrenia. | Qualitative (open-ended interviews) | Home, public place, psychiatric clinic | schizophrenia | NR | 45 | Hermeneutical analysis | NR | 8 |
| De et al., 2013 | India | To explore subjective experience with bizarre delusions | Qualitative (retrospective records) | Tertiary care hospital | schizophrenia | DSM-IV | 1952 | Thematic analysis | 30,59 (9,47) | 8 |
| Dickson et al., 2016 | UK | To explore the association between childhood experiences and paranoia | Qualitative (semi-structured interviews) | NHS and Early Intervention Psychosis service | SSDs | NR | 7 | Interpretative phenomenological analysis (IPA) | 34 | 8 |
| Fascendini et al., 2025 | Italy | To explore the subjectivity of delusions in schizophrenia | Qualitative (semi-structured interviews) | Psychiatry Unit of University Department | schizophrenia | DSM 5 | 11 | Interpretative phenomenological analysis (IPA) | 34.9 (10.79) | 9 |
| Feyaerts et al., 2021 | Belgium | To explore the experience of delusions in schizophrenia | Qualitative (semi-structured interviews) | Psychiatric hospital services | SSDs | DSM 5 | 18 | Interpretative phenomenological analysis (IPA) | 39.7 (13.5) | 9 |
| Fierro et al., 2018 | Colombia | To explore delusion of control in subjects with SSDs spectrum disorders | Qualitative (semi-structured interviews) | NR | SSDs | DSM 5 | 7 | Phenomenological analysis | 35.9 (14.8) | 8 |
| Flanagan et al., 2012 | USA | To explore subjective experience of schizophrenia in relation to DSM-IV-TR criteria | Qualitative (interviews) | Mental health centre | SSDs | DSM-IV-TR | 17 | Interpretative phenomenological analysis (IPA) | 47,65 (6,96) | 9 |
| Gee et al., 2019 | UK | To explore subjects' experience with negative symptoms | Qualitative (in-depth interviews) | Early Intervention in Psychosis services | SSDs | ICD-10 | 24 | Thematic analysis | 27.6 (4.15) | 9 |
| Gordon et al., 2023 | USA | To explore differences in paranoia between black and white adults | Qualitative (semi-structured archival interviews) | Community out subjects | SSDs | DSM 5 | 21 | Thematic analysis | NR | 9 |
| Hansen et al., 2022 | USA | To explore motivation and negative symptoms in SSDs | Qualitative (semi-structured interviews) | Community program | SSDs | DSM 5 | 21 | Thematic analysis | 41.43 (13.04) | 9 |
| Hirschfeld et al., 2005 | UK | To explore psychotic experiences in young men | Qualitative (semi-structured interviews) | Clinical settings | schizophrenia | NR | 6 | Grounded theory | 23.33 (3.5) | 9 |
| Isham et al., 2021 | UK | To explore subjects' experience with grandiose delusions | Qualitative (semi-structured interviews) | National Health Services (NHS) | SSDs, bipolar disorder | NR | 8 | Grounded theory | NR | 10 |
| Jakes et al., 2004 | NR | To explore the relation between themes of delusions and life problems | Qualitative (semi-structured interviews) | Clinical psychology service | SSDs, bipolar disorder | NR | 18 | Interpretative phenomenological analysis | NR | 6 |
| Jarosinski, 2008 | USA | To explore subjective experience with hallucinations | Qualitative (semi-structured interviews) | Two recruitment sites (not specified) | SSDs | NR | 12 | Hermeneutical analysis | 50 | 9 |
| Johnson et al., 1997 | Canada | The purpose of this qualitative study was to increase the understanding of the experiences of individuals with thought disorders, which precede incidents of aggression. | Qualitative (interviews) | Tertiary care hospital and general hospital | SSDs, bipolar disorder | NR | 11 | Content analysis | NR | 8 |
| Jones et al., 2016 | USA | To examine how individuals with psychotic spectrum disorders navigate cultural and clinical tensions between reality and delusion, and between sacred and secular beliefs. | Qualitative (semi-structured in-depth interviews) | Community mental health and services user advocacy settings | SSDs | NR | 15 | Grounded theory | NR | 9 |
| Judge et al., 2008 | USA | To explore how individuals recognize psychosis | Qualitative (retrospective semi-structured interviews) | Public outpatients’ psychiatric clinic | SSDs | DSM-IV | 15 | Grounded theory and content analysis | 24.4 (5.28) | 9 |
| Knudson & Coyle, 2002 | UK | To explore personal experiences of individuals who hear voices | Qualitative (semi-structured interviews) | NR | schizophrenia | DSM-IV | 2 | Interpretative phenomenological analysis (IPA) | 24.63 (4.03) | 7 |
| Larsen, 2004 | Denmark | To explore subjective experience with first episode psychosis | Qualitative (semi-structured interviews) | Community mental health early intervention program | SSDs | ICD-10 | 15 | Thematic analysis | NR | 6 |
| Löfs & Rasmussen, 2025 | Denmark | To explore the phenomenology of auditory and visual hallucinations in schizophrenia | Mixed-methods (semi-structured interviews and PANSS, GAF, BSABS, EASE) | Non-acute wards and outpatient facilities | schizophrenia spectrum | ICD-10 | 32 | Phenomenological analysis | NR | 8 |
| Marshall et al., 2020 | UK | To explore body image in psychotic subjects with delusions | Qualitative (semi-structured interviews) | Home or mental health clinic | SSDs | NR | 12 | Interpretative phenomenological analysis (IPA) | 43 (10.6) | 10 |
| Moernaut et al., 2018 | Belgium | To explore hallucinations through a Lacanian framework | Qualitative (semi-structured interviews) | Hospital | schizophrenia | DSM 5 | 10 | Thematic analysis | 43.89 (16.08) | 9 |
| Moernaut et al., 2022 | Belgium | To explore subjective experience with negative symptoms in psychosis | Qualitative (semi-structured interviews) | Wards in two psychiatric hospitals | SSDs | DSM 5 | 12 | Interpretative phenomenological analysis (IPA) | NR | 9 |
| Møller & Husby, 2000 | Norway | To explore core dimensions of SSDs | Qualitative (in-depth interviews) | SSDs unit | SSDs | DSM-III-R, DSM-IV | 19 | Content analysis | 22.4 (3.1) | 9 |
| Ng et al., 2012 | Hong Kong | To explore subjects' coping strategies with auditory hallucinations | Qualitative (semi-structured interviews) | Interview rooms at two non-governmental organizations | schizophrenia | NR | 20 | Thematic analysis | NR | 9 |
| Noroozi et al., 2025 | Australia | To explore autonomy in voice-hearers | Qualitative (semi-structured interviews) | Swinburne Voices Clinic | schizophrenia spectrum, bipolar disorder | NA | 3 | Interpretative phenomenological analysis (IPA) | NR | 9 |
| Pietkiewicz et al., 2021 | Poland | To explore delusions of possession in SSDs | Qualitative (semi-structured interviews) | Research centre | schizophrenia | ICD-10 | 4 | Interpretative phenomenological analysis (IPA) | 26.5 (4.36) | 9 |
| Rhodes & Healey, 2017 | UK | To explore subjective experience of childhood abuse in psychosis | Qualitative (semi-structured interviews) | Hospital based psychology department | SSDs, bipolar disorder | NR | 7 | Interpretative phenomenological analysis (IPA) | 37.75 (9.7) | 9 |
| Rhodes & Jakes, 2004 | NR | To explore subjective experiences of delusions | Qualitative (CBT session) | Community services | SSDs, depression with psychotic features, personality disorder | NR | 20 | Interpretative phenomenological analysis | NR | 8 |
| Rhodes et al., 2016 | UK | To explore psychosis in refugees | Qualitative (semi-structured in-depth interviews) | NR | Psychosis NOS, PTSD | NR | 2 | Interpretative phenomenological analysis (IPA) | 34.71 (6.34) | 9 |
| Rieben et al., 2013 | Switzerland, Canada | To explore subjective experiences of religious content in delusions | Qualitative (semi-structured interviews) | Psychiatric facilities and Assertive Community Treatment | SSDs | ICD-10 | 62 | Grounded theory | 42 (11) | 9 |
| Shepherd et al., 2012 | USA | To explore subjective experience of SSDs in older subjects | Qualitative (semi-structured interviews) | University research centre | schizophrenia | NR | 32 | Grounded theory | 55.7 (4.1) | 9 |
| Simmons et al., 2024 | USA | To explore subjects' experience of SSDs during participation in a RCT | Qualitative (semi-structured interviews) | Clinical trial sites, patient's home (videoconference) | schizophrenia | DSM 5 | 41 | Thematic analysis | 44.2 (9.5) | 9 |
| Skourteli et al., 2025 | Grece | To explore the lived experience of psychosis in patients and caregivers | Qualitative (semi-structured interviews) | Day Centre for Psychosocial Rehabilitation | schizophrenia spectrum | ICD-10 | 4 | Thematic analysis | 33.75 (8.62) | 10 |
| Startup et al., 2015 | UK | To explore persecutory delusions | Qualitative (semi-structured interviews) | Local mental health base or patient's home | SSDs | NR | 8 | Interpretative phenomenological analysis (IPA) | 44.25 (14.95) | 9 |
| Stephensen et al., 2024 | Denmark | To explore the experience of double reality in SSDs subjects | Qualitative (semi-structured interviews) | Inpatient and outpatient psychiatric clinics | SSDs | ICD-10 | 25 | Thematic analysis | 30.7 (11.3) | 9 |
| Stopa et al., 2013 | UK | To explore the experience of threat in social phobia and paranoia | Qualitative (semi-structured interviews) | Hospital setting | schizophrenia, social phobia | DSM-IV-TR | 9 | Thematic analysis | 37.4 (12.7) | 8 |
| Taylor et al., 2020 | UK | To explore core beliefs in psychosis | Qualitative (semi-structured interviews) | Early Intervention Services and Community Mental Health Teams | SSDs | ICD-10 | 20 | Thematic analysis | 31.49 (9.0) | 10 |
| Toh et al., 2020; Toh et al., 2024 | Australia | To explore auditory and visual hallucinations in psychosis | Mixed-methods (semi-structured in-depth interview and MINI, PANSS, YMRS, BAI, BDI, MSI-BPD) | Public and private mental health services, community venues, online advertising | SSDs, bipolar disorder, major depressive disorder | NR | 76 | Thematic analysis | NR | 10 |
| Topor & Di Girolamo, 2010 | France | To explore how people with psychosis manage crisis | Qualitative (focus group) | User organization | SSDs | NR | 15 | Grounded theory | NR | 7 |
| Upthegrove et al., 2016 | UK | To explore auditory verbal hallucinations in first-episode psychosis | Qualitative (in-depth interviews using diary and photo-elicitation) | Early Intervention service | SSDs | ICD-10 | 25 | Content analysis | 26 | 9 |
| Wahyuni et al., 2021 | Indonesia | To explore subjects' experience with hallucinations | Qualitative (semi-structured interviews) | Psychiatric asylum | schizophrenia | NR | 15 | Colaizzi's method | 26 | 8 |
| Wood et al., 2015 | UK | To explore cognitive impairment in SSDs | Mixed-methods (cognitive tests for inclusion and semi-structured interviews) | Suburban NHS trust | schizophrenia | NR | 8 | Interpretative phenomenological analysis (IPA) | 44 | 10 |
| Xanthopoulou et al., 2022 | UK | To explore subjects, caregivers and mental health practitioners' experiences with social isolation | Qualitative (focus groups) | Community mental health services | SSDs | ICD-10 | 32 | Inductive thematic analysis | 41 (9.8) | 9 |
| Yttri et al., 2020 | Denmark | To explore auditory verbal hallucinations in SSDs | Qualitative (semi-structured interviews) | University department of psychiatry | schizophrenia | ICD-10 | 20 | Phenomenological analysis | 32 (10.4) | 8 |

**Table C.2** Overview of themes with corresponding explanations identified in the thematic analysis for general illness experience (I).

| **Theme** | **Sub-theme** | **Explanation** |
| --- | --- | --- |
| (a) Burden and impact of illness | Social impairment | Patients reported losing social connections, leading to isolation. |
|  | Physical and cognitive impairment | Fatigue and reduced cognitive energy were common barriers to daily functioning. |
|  | Disruption of life roles | Many described abandoning life goals (e.g., parenthood) due to illness. |
|  | Motivational loss | The illness itself was experienced as blocking ambitions and making goal-setting impossible |
| (b) Isolation, stigma, desire to belong | Stigma and shame | Patients felt more comfortable disclosing depression than schizophrenia due to stigma. |
|  | Damaged self-image | Described themselves as “weak” or “useless,” reflecting internalised stigma. |
|  | Distrust and broken dialogue | Reported being silenced in hospital settings, unable to express their thoughts. |
|  | Emotional disconnection | Felt detached from others, even in crowds, describing loneliness and alienation. |
| (c) Relationship with drug, clinicians and health care system | Ambivalence toward drugs | Acknowledged medication’s necessity but disliked reliance on it. |
|  | Experience of coercion | Some recalled forced treatment (e.g., chemical restraint) as traumatic. |
|  | Negotiating agency | Patients sometimes agreed to comply voluntarily but emphasised lack of autonomy. |
|  | Lack of information | Felt uninformed and overlooked, with doctors too busy to explain or listen. |
| (d) Disruption of self, reality and narrative reconstruction | Loss of engagement | Reported inability to connect with others or activities. |
|  | Fragmentation of self | Felt broken or disintegrated, describing self as “in pieces.” |
|  | Altered reality | Questioned the reality of past experiences, struggling to distinguish real from unreal. |
|  | Struggle in meaning-making | Found it difficult to articulate or make sense of experiences. |
|  | Narrative reconstruction and existential reframing | Some reframed their identity, seeing themselves as people with desires and worth beyond the illness. |
| (e) Support and coping strategies | Social support | Relationships and encouragement from others were protective and meaningful. |
|  | Functional coping strategies | Talking to someone or engaging in activities helped manage distress. |
|  | Dysfunctional coping strategies | Withdrawal when overwhelmed or substance misuse to deal with symptoms. |

**Table C.3** Overview of themes with corresponding explanations identified in the thematic analysis (II).

| **Dimension** | **Symptom** | **Themes** | **Example quotes** |
| --- | --- | --- | --- |
| Disorders of perceptions | Hearing voices | (1) Phenomenological characteristics; (2) Fear, sadness, unpleasant emotions; (3) Pleasant emotions; (4) Coping and meaning-making | (1) “She’s like, she’s from England, she just won’t tell me where” (Alderson-Day et al., 2021); (2) “The voices just make me tired all the time, they never stop, only when I go to sleep.” (Butcher et al., 2020); (3) “I would have voices that I loved. And I loved connecting with them and I cared about them so much.” (Clements et al., 2020); (4) “I just thought it was something I experienced, that it was normal and the way I experienced the world. When I got these questions: “do you hear voices,” I said, “but I do not know if I do” and they explained by saying “do you hear something you feel others cannot hear?” And yes, I did, and I thought, “well, it must be “voices” that I can hear” (Yttri et al., 2020) |
|  | Other auditory hallucinations | (1) Human, animal or environmental sounds | (1) “I could hear like noises, like of a rustle outside the door.” (Rhodes & Jakes, 2004) |
|  | Visual hallucinations | (1) Threatening and violent behaviour; (2) Truth and subjugation; (3) Blurred and dreamy reality | (1) “I feel vulnerable to people attacking me…I've had images of being stabbed, ermm, being decapitated, and it's been friend's faces which have done it. (Taylor et al., 2020)”; (2) “I know better, and I know everything I was doing. I couldn’t walk straight and all I wanted to do was sit out in the rain and all of that. And I know every  thing I was doing, but I just couldn’t help it.” (Jarosinski et al., 2016); (3) “I probably fell asleep and I wake up and I swear I see a figure in a cloud or something and I see a gun pointed at me. And it said go back where you belong.” (Jarosinski, 2008) |
|  | Bodily hallucinations | (1) Intrusion; (2) Altered body parts; (3) Painful sensations | (1) “There were worms inside the nose and the nerves were eaten up, everything became sick.” (De et al., 2013); (2) “Sometimes I might go down my tonsils and all . . . they get bigger—kind of bigger—sometimes they go down but I can’t say what kind of disease it is.” (Jarosinski, 2008); (3) “When I started doing, feeling those burning pains all over my body, as if I was being burnt.” (Rhodes & Jakes, 2004) |
|  | Other hallucinations | (1) Different reality; (2) Meaningful experience; (3) Coping strategies; (4) Suicidality, distress, unpleasant emotions | (1) “It [hallucinatory experience] felt as if it exceeded consciousness, like it ‘bubbled over.’ You can no longer describe it, because it is so.. it was so.. it was so.. so wild and it was so beyond, it was so beyond (…) It’s extremely difficult to describe (…) like a pure sensing without logical thought.” (Stephensen et al., 2024); (2) “As for me, my hallucinations are real important. I don’t see them as a problem; instead, I need them. Some actually nurture me and don’t devastate me.” (Topor & Di Girolamo, 2010); (3) “The actions taken by them to overcome the hallucination are closing their ears, having a chat, scolding the hallucination, waking back and forth, being angry, wrecking furniture, doing what the hallucination commands to do, answering the hallucination, laughing, closing their eyes, taking medicine, doing spiritual activities, and doing outdoor activities and running” (Wahyuni et al., 2021); (4) “Umm, like I want to commit suicide and don’t want to live, don’t want to be this way, I want to be like somebody else, I want to be different. Ah, I, some time I sleeps at night and I toss and turn, then I wake up dreaming and ah, wake up scared, some body is scaring me, over me, trying to do something to me. And I wakes up being scared, and like a lot and I end up crying in the middle of the night when I’m asleep.” (Jarosinski, 2008) |
| Delusions | Delusion of grandiosity | (1) Transformed identity and superhuman status; (2) Social consequences and risky situations; (3) Power, agency, protective purposes; (4) Repetitive thinking | (1) “I was convinced that others would consider me as a figure of Jesus, that I discovered heaven, and that I proved the existence of the supernatural.” (Feyaerts et al., 2021); (2) “I was on the lamppost on the bridge, sitting on top of it.[...] I just wanted to see the horizon line. I was literally just obsessed with space and that.” (Isham et al., 2021); (3) “People just kept staring at me wherever I went [...]’. Interviewer: ‘What did you conclude from that?’ Stephen: ‘That I was something powerful’.” (Isham et al., 2021); (4) “I spent a lot of time thinking about it, not that much time like researching about it. Just thinking, thinking about it and feeling I would get the right answers myself without actually looking it up.” (Isham et al., 2021) |
|  | Delusion of guilt | (1) Religion and sin; (2) Compulsions | (1) ‘‘Everything that is pleasure I consider it as a sin.’’ (Rieben et al., 2013); (2) ‘‘I need to lie to others in order to be able to have time to do these rituals.’’ (Rieben et al., 2013) |
|  | Delusion of persecution | (1) Surveillance and control; (2) Distrust and fear of harm; (3) Overwhelmed and suicidal | (1) “Well I suppose it was like the big brother thing but I had no consent...I thought everything was a big plot and people were just watching me”. (Campbell & Morrison, 2007); (2) “He said, ‘would you like something to eat?’ I went, ‘mmm’, like that, I did not answer him really. And he come in and he went to give me some toast, and I went to bite it, and I went, I was so paranoid I said, ‘you poisoned this, I am not going to eat it’. He is like tearing his hair out, he could not understand it.” (Hirschfeld et al., 2005); (3) “I was just feeling stressed, angry and unhappy... I tried to take an overdose once... of sleeping tablets” (Hirschfeld et al., 2005) |
|  | Delusion of reference | (1) Emotional pain; (2) Social withdrawal and unusual behavior | (1) “I thought they were talking about me in the other room ...I thought he was making plans to make me look stupid.” (Stopa et al., 2013); (2) “Uh, wear a pair of sunglasses and hide away.” (Stopa et al., 2013) |
|  | Delusional ideas | (1) Different reality | (1) “I’ve always lived in two parallel worlds.. Meaning that I live in the world everybody else does, where we know that the table is a table, and then in my own world, where I have visions and hear voices. But my sense of reality is intact. I know that you can’t see and hear what I can see. I can easily keep them apart.” (Stephensen et al., 2024) |
|  | Delusional mood | (1) Different reality; (2) Existential revelations and spiritual dimensions; (3) Fear, loss of control, self-harm | (1) “Earth used to be everything. But now we’ve been to Mars, Earth has become a little circle in the distance. Do you get it?” (Feyaerts et al., 2021). (2) “I lived between two realities. Much of our time we are here on earth taking care of our daily business. But on the other hand, there is this question of the purpose of life, of God and the angels. I just couldn’t get a grip on the situation.” (Feyaerts et al., 2021). (3) “What was left of myself had to do something powerful, so I stabbed myself.” (Moller & Husby, 2020) |
|  | Other delusions | (1) Religious content; (2) Illness-related and somatic content; (3) Identity transformation; (4) Control and manipulation | (1) “The devil got into my body. He came like air, entering through an ear and staying inside the head. During that time, he did me so much harm because he tried to steal my soul and control me. It took me a lot of effort to expel him, although sometimes he left by himself. I used plugs in my ears to prevent him from entering, but it did not work. I couldn’t fight someone so powerful.” (Fierro et al., 2018); (2) “Girls have cancer in their mouth and if I kiss them I will get surely ill (AIDS) and might vomit out.” (De et al., 2013); (3) “I am a dog” and ran here and there like a dog acting on his beliefs. (De et al., 2013); (4) He started thinking someone else had invaded his body, and the recent memory of his cousin’s death convinced him it was his cousin’s spirit who possessed him. In trying to make the experience comprehensible to himself and others, especially the odd character of depersonalization, the patient concluded that he was possessed by his cousin, who intended to harm him. (Fierro et al., 2018) |
| Worries and compulsions | Suspiciousness | (1) Paranoia and fear | (1) “[I’m] nervous and paranoid that people are listening.” (Flanagan et al., 2012) |
| Ego boundaries disturbances | Thought broadcasting | (1) Loss of boundaries | (1) “And then he said, ‘It’s fun, isn’t it, that they can hear everything?’ Marc was as it were telling me, but he wasn’t really saying anything like that.” (Moernaut et al., 2018) |
|  | Depersonalization | (1) Emptiness and disconnection | (1) “You cannot connect with uhm, with what you [feel] inside, uhm, there is no experience and you are confronted with a limit, like ‘that is what I feel’, or, yeah, physically, but you can no longer envision it at all”. (Moernaut et al., 2022) |
|  | Derealization | (1) Feeling stuck in a dream; (2) Estrangement and disconnection | (1) “I was in a sort of dream world and when I looked at the medical staff, they were just sitting there as some sort of machines, they only seemed to move when I looked at them.” (Feyaerts et al., 2021); (2) “Missing a fundamental skill to connect to the world” (Moernaut et al., 2022) |
| Formal thoughts disorders | Pressured thinking | (1) Fear | (1) “I start crying…because the thoughts are so scary and bad and you don’t know what’s going on in your head…All you can do is cry.” (Flanagan et al., 2012) |
|  | Inhibited thinking | (1) Emptiness | (1) “That emptiness, is actually best compared with, well yeah, actual emptiness. There is absolutely nothing in your head and, well yeah, you’re not thinking” (Moernaut et al., 2022) |
|  | Rumination | (1) Psychological and behavioural distress; (2) Intrusiveness | (1) "It worries me all the time... all the time... always worried about it." (Startup et al., 2015); (2) "...I’m just thinking about it constantly ...I can’t put my mind onto things I want to think about, I just think about that all the time." (Stopa et al., 2013) |
|  | Flight of ideas | (1) Inability to control thoughts | (1) “Thoughts went in all directions” (Moller et al., 2000) |
|  | Incoherence/Derailment | (1) Disconnected, illogical | (1) “I started answering like one and one equals two. I was going like this making arrows and pluses. Your brain just can’t…I couldn’t even think to add it up. It was like I was losing my mind.” (Flanagan et al., 2012) |
|  | Thought blocking | (1) Confusion | (1) “It’s like confusion. I can’t say what I want to say because my head goes blank.” (Flanagan et al., 2012) |
| Disturbances of affect | Loss of feelings | (1) Numbness | (1) “Yeah. Erm numb. Blocked. My head was sort of blocked. I couldn't think, therefore couldn't speak, because I didn't know what to say.” (Gee et al., 2019) |
|  | Blunted affect | (1) Reduced emotional expression; (2) Withdrawal | (1) “I really don’t want to be emotionally flattened. I really want to keep my brains, uhm, my mind. I’m still in my right mind, am I not? A little flattened… do I appear flattened to you?” (Moernaut et al., 2022); (2) “I don’t do it [express myself emotionally]. Even with my clinician I don’t do it because I don’t want people to think I’m crazy, you know. So a lot of things I harbour on the inside of me.” (Flanagan et al., 2012) |
|  | Depressed mood | (1) Burden and negative self-perception; (2) Illness-related depression | (1) “I was in shock…I was very embarrassed, humiliated, disgusted, and depressed over my illness.” (Flanagan et al., 2012); (2) "I started sinking into a deeper depression...it got to the point where I didn’t want to watch TV, eat, like it got that bad." (Boydell et al., 2003) |
|  | Irritability | (1) Hurtful sensitivity | (1) “Oh my god. Sensitivity, hurtful. I get over anxious sometimes. I get very angry in certain situations. I’m easy to upset.” (Flanagan et al., 2012) |
|  | Affective incontinence | (1) Feeling vulnerable; (2) Anxiety and fear | (1) “I feel sad, sensitive. I break just like glass if you say anything bad to me.” (Flanagan et al., 2012); (2) “Can’t breathe properly, feel suffocated” (Stopa et al., 2013) |
| Disorders of consciousness | Expanded consciousness | (1) Intensified perceptions; (2) Mystical experiences | (1) “I had a sort of heightened perception; I saw connections everywhere, connections which I alone saw, for example, on the doors of the psychiatric ward. The semantics of words revealed a hidden meaning.” (Feyaerts et al., 2021); (2) “I gained a new form of consciousness, discovered a new world which others couldn’t follow. I was everywhere, I thought I discovered the key for true love.” (Feyaerts et al., 2021) |
| Additional psychopathological items | Hearing one's own thoughts | (1) Inner voice | (1) “Basically what it is, is my thoughts, in the form of a voice…just like talking inside my head.” (Flanagan et al., 2012) |
|  | Acceleration of thinking | (1) Social impairment; (2) Cognitive overload | (1) “My thoughts were so numerous that I didn't manage talking to people.” (Moller et al., 2000); (2) “...your thoughts are racing...” (Stopa et al., 2013) |
| Disorders of drive | Inhibition of drive | (1) Feeling shut | (1) “I can’t function. I’ll shut right down, like I’m—I could be washing a dish sometime. It falls. I just—I just can’t work.” (Flanagan et al., 2012) |
|  | Lack of drive | (1) Zombie-like state; (2) External hindrance | (1) “I wasn't moving, I was sitting down… I wasn't talking. I was just like, you know, like a zombie, just sitting there…I'd just sit down and not interact with anyone.” (Gee et al., 2018); (2) “It’s like a nightmare... living in a nightmare...you can’t, you try and push yourself but you can’t ...you cannot do It ...Summat holding you back all time ...and it feels like I’m being pulled back ...you’re not going forward or anywhere like that.” (Butcher et al., 2020) |
|  | Mannerism | (1) Feeling judged | (1) “I was half bent over, and walked in this way. I couldn’t control it and people commented about it. They said I must be ill or fucked up. They paid attention to me because I behaved weird.” (Pietkiewicz et al., 2021) |
| Other disturbances | Social withdrawal | (1) Seeking solitude; (2) Fear of stigma | (1) "I do not communicate much… I know that I am more solitary than normal and that it is negative in people's eyes. I need to withdraw, to be alone occasionally." (Corin 1998); (2) “I wasn’t feeling [like] myself, and things were strange, and I guess I was reluctant to tell people. It was kind of outrageous what was going on, and it was hard to put into words because it was so complicated… so I did pull back.” (Judge et al., 2008) |
|  | Self-harm | (1) Non-acceptance | (1) “I cut my face ‘cos I wanted to look different, I cut my face with a razor.” (Marshall et al., 2020) |
|  | Aggressiveness | (1) Environmental and relational triggers; (2) Self-regulation attempts; (3) Frustration and powerlessness | (1) “There was a barbecue outside....It made me furious because I was outside the barbecue....I wanted to be there....Nobody would let me go. I was locked in.” (Johnson et al., 1997); (2) “I do art work to relax....Do something to take my mind off the frustrations....Drawing, do some drawing. Read a book. Do something to keep my mind off of that.” (Johnson et al., 1997); (3) “I missed the interview, so I went and told her, ‘Sorry I missed the interview, I fell asleep’....She insinuated that I was lying, and I got very upset. So I was, had my electric shaver, and I was just ready to take a shave. And I threw it on the floor, broke it.” (Johnson et al., 1997) |
| Disturbances of attention and memory | Memory impairment | (1) Trying coping strategies | (1) “I wondered if I have to take gingko biloba… it’s good for the memory.” (Wood et al., 2015) |
|  | Disturbed concentration | (1) Impairment in daily living | (1) “It’s hard, you know, to concentrate. I used to be able to read like 50 pages of a book every day and f finish the book in 7 days. Now I read like two pages and I don’t read the book no more.” (Flanagan et al., 2012) |

**Appendix D: CASP scores**

**Table D.1** Detailed CASP scores for each individual item

| **Authors** | **1.Was there a clear statement of the aims of the research?** | **2. Is a qualitative methodology appropriate?** | **3.Was the research design appropriate to address the aims of the research?** | **4. Was the recruitment strategy appropriate to the aims of the research?** | **5. Was the data collected in a way that addressed the research issue?** | **6. Has the relationship between researcher and participants been adequately considered?** | **7. Have ethical issues been taken into consideration?** | **8. Was the data anot reportedlysis sufficiently rigorous?** | **9. Is there a clear statement of findings?** | **10. How valuable is the research?** |
| --- | --- | --- | --- | --- | --- | --- | --- | --- | --- | --- |
| (Alderson-Day et al., 2021) | no | yes | yes | yes | yes | not reported | yes | yes | yes | yes |
| (Boydell et al., 2003) | yes | yes | yes | yes | yes | not reported | yes | yes | yes | yes |
| (Butcher et al., 2020) | yes | yes | yes | yes | yes | yes | yes | yes | yes | yes |
| (Campbell & Morrison, 2007) | yes | yes | yes | yes | yes | not reported | yes | yes | yes | yes |
| (Cassaniti et al., 2025) | yes | yes | yes | yes | yes | not reported | yes | yes | yes | yes |
| (Chernomas et al., 2000) | yes | yes | yes | yes | yes | not reported | not reported | no | yes | yes |
| (Clements et al., 2020) | yes | yes | yes | yes | yes | yes | yes | yes | yes | yes |
| (Corin, 1998) | yes | yes | yes | yes | yes | not reported | not reported | yes | yes | yes |
| (De et al., 2013) | yes | yes | yes | yes | yes | not reported | yes | no | yes | yes |
| (Dickson et al., 2016) | yes | yes | yes | yes | yes | not reported | not reported | yes | yes | yes |
| (Fascendini et al., 2025) | yes | yes | yes | yes | yes | not reported | yes | yes | yes | yes |
| (Feyaerts et al., 2021) | yes | yes | yes | yes | yes | not reported | yes | yes | yes | yes |
| (Fierro et al., 2018) | yes | yes | yes | yes | yes | not reported | yes | not reported | yes | yes |
| (Flanagan et al., 2012) | yes | yes | yes | yes | yes | not reported | yes | yes | yes | yes |
| (Gee et al., 2019) | yes | yes | yes | yes | yes | not reported | yes | yes | yes | yes |
| (Gordon et al., 2023) | yes | yes | yes | yes | yes | not reported | yes | yes | yes | yes |
| (Hansen et al., 2022) | yes | yes | yes | yes | yes | not reported | yes | yes | yes | yes |
| (Hirschfeld et al., 2005) | yes | yes | yes | yes | yes | not reported | yes | yes | yes | yes |
| (Isham et al., 2021) | yes | yes | yes | yes | yes | yes | yes | yes | yes | yes |
| (Jakes et al., 2004) | yes | yes | yes | yes | yes | not reported | not reported | yes | no | no |
| (Jarosinski, 2008) | yes | yes | yes | yes | yes | not reported | yes | yes | yes | yes |
| (Johnson et al., 1997) | yes | yes | yes | yes | yes | not reported | not reported | yes | yes | yes |
| (Jones et al., 2016) | yes | yes | yes | yes | yes | no | yes | yes | yes | yes |
| (Judge et al., 2008) | yes | yes | yes | yes | yes | not reported | yes | yes | yes | yes |
| (Knudson & Coyle, 2002) | yes | yes | yes | yes | yes | not reported | not reported | yes | yes | no |
| (Larsen, 2004) | yes | yes | yes | not reported | yes | not reported | not reported | yes | no | yes |
| (Löfs & Rasmussen, 2025) | yes | yes | yes | yes | yes | not reported | yes | no | yes | yes |
| (Marshall et al., 2020) | yes | yes | yes | yes | yes | yes | yes | yes | yes | yes |
| (Moernaut et al., 2018) | yes | yes | yes | yes | yes | not reported | yes | yes | yes | yes |
| (Moernaut et al., 2022) | yes | yes | yes | yes | yes | no | yes | yes | yes | yes |
| (Møller & Husby, 2000) | yes | yes | yes | yes | yes | not reported | yes | yes | yes | yes |
| (Ng et al., 2012) | yes | yes | yes | yes | yes | not reported | yes | yes | yes | yes |
| (Noroozi et al., 2025) | yes | yes | yes | yes | yes | not reported | yes | yes | yes | yes |
| (Pietkiewicz et al., 2021) | yes | yes | yes | yes | yes | not reported | yes | yes | yes | yes |
| (Rhodes & Healey, 2017) | yes | yes | yes | yes | yes | not reported | yes | yes | yes | yes |
| (Rhodes & Jakes, 2004) | yes | yes | yes | yes | yes | no | not reported | yes | yes | yes |
| (Rhodes et al., 2016) | yes | yes | yes | yes | yes | not reported | yes | yes | yes | yes |
| (Rieben et al., 2013) | yes | yes | yes | yes | yes | not reported | yes | yes | yes | yes |
| (Shepherd et al., 2012) | yes | yes | yes | yes | yes | not reported | yes | yes | yes | yes |
| (Simmons et al., 2024) | yes | yes | yes | yes | yes | not reported | yes | yes | yes | yes |
| (Skourteli et al., 2025) | yes | yes | yes | yes | yes | yes | yes | yes | yes | yes |
| (Startup et al., 2015) | yes | yes | yes | yes | yes | not reported | yes | yes | yes | yes |
| (Stephensen et al., 2024) | yes | yes | yes | yes | yes | not reported | yes | yes | yes | yes |
| (Stopa et al., 2013) | yes | yes | yes | yes | yes | not reported | not reported | yes | yes | yes |
| (Taylor et al., 2020) | yes | yes | yes | yes | yes | yes | yes | yes | yes | yes |
| (Toh et al., 2020) | yes | yes | yes | yes | yes | not reported | yes | yes | yes | yes |
| (Toh et al., 2024) | yes | yes | yes | yes | yes | yes | yes | yes | yes | yes |
| (Topor & Di Girolamo, 2010) | yes | yes | yes | yes | yes | not reported | not reported | yes | yes | no |
| (Upthegrove et al., 2016) | yes | yes | yes | yes | yes | not reported | yes | yes | yes | yes |
| (Wahyuni et al., 2021) | yes | yes | yes | yes | not reported | not reported | yes | yes | yes | yes |
| (Wood et al., 2015) | yes | yes | yes | yes | yes | yes | yes | yes | yes | yes |
| (Xanthopoulou et al., 2022) | yes | yes | yes | yes | yes | not reported | yes | yes | yes | yes |
| (Yttri et al., 2020) | yes | yes | yes | yes | yes | not reported | yes | no | yes | yes |

**Table D.2** Detailed CASP comments for each individual item

| **Authors** | **Item 1** | **Item 2** | **Item 3** | **Item 4** | **Item 5** | **Item 6** | **Item 7** | **Item 8** | **Item 9** | **Item 10** |
| --- | --- | --- | --- | --- | --- | --- | --- | --- | --- | --- |
| (Alderson-Day et al., 2021) | We aimed to investigate personification in individuals’ early voice-hearing experiences. | A qualitative methodology is appropriate to explore social qualities of hallucinations | Participants took part in a semi structured interview with  1 of 2 interviewers trained in clinical, phenomenological,  and qualitative health interviewing. | A pragmatic sample of  40 participants was recruited to enable in-depth qualitative interviewing and analysis, exploratory quantitative analysis, and longitudinal follow-up. | All interviews were recorded and then professionally  transcribed for analysis | NR | All procedures  were approved by a local National Health Service (NHS)  Research Ethics Committee. | Content analysis and inductive thematic analysis were used to derive a coding  frame that permitted direct comparison with prior phenomenological surveys of AVH, while also allowing  for a nuanced analysis of the specific qualities of the data  collected. | Examples of quotes are reported for qualitative results | Clinical and demographic characteristics of the sample are reported so |
| (Boydell et al., 2003) | The purpose of this study is to explore the subjective experience of motivation as revealed in the narratives of persons diagnosed with schizophrenia and the meanings individuals attached to this experience. | A qualitative methodology is appropriate to explore subjective experiences | A qualitative descriptive approach was used to focus on the participants’ experiences and interpretation of the subject matter | Three individuals were recruited from a self-help organization, two were recruited from a first-episode hospital clinic and one was recruited through the participant’s place of employment. | All interviews were audiotaped, transcribed verbatim and converted into the format required | NR | The study received Institutional Ethics Review Board approval prior to commencing. | Analysis of the narrative text followed the seven stages as outlined in the work of Diekelmann (1992). | Findings are clearly discussed and divided into themes | The diversity of the sample enhances the generalizability of the findings. |
| (Butcher et al., 2020) | This study sought to examine, using qualitative methods, how people living with a diagnosis of schizophrenia subjectively experience negative symptoms. | A qualitative methodology is appropriate to examine how people live with a diagnosis of schizophrenia | Semi-structured interviews were carried out with individuals with a diagnosis of schizophrenia who were experiencing negative symptoms. | The sample was recruited from community and inpatient National Health Service mental health settings in the United Kingdom. | Each interview was transcribed verbatim by the lead author with all data anonymized as much as possible, and all data were kept confidential and stored securely | This study was conducted in a rigorous and high-quality manner. Reading and discussion of each transcript by the research team ensured that the process was iterative and transparent. It is acknowledged that each author’s experiences inevitably shape data analysis. | This study was reviewed by a North West Ethics Committee and was carried out from April 2017 to July 2018. | Thematicdataanalysiswaschosenasitisaflexibleprocesswhichenablestheexploration of rich data in an efficient method. | Main themes are summarized into a figure; supporting quotes are provided | Sample size is sufficient for a qualitative study. The heterogeneity permit data generalizability |
| (Campbell & Morrison, 2007) | The main aim of the study was to find out about people’s subjective experience of paranoia; this will be done by comparing both a patient’s and non-patient’s discourse on the topic. | A qualitative methodology is appropriate to explore | Participants were interviewed and the data were then transcribed and analysed using interpretative phenomenological analysis (IPA). | Five of these participants were recruited from community mental health teams and one was approached by the researcher subsequent to participating in a prior study. | All of the interviews were tape recorded in order to facilitate analysis and to allow the dialogue to continue uninterrupted | NR | The local NHS Research Ethics committee approved the study. | The data was subjected to IPA. | Emerging themes are summarized in a table | Quotes from patients are clearly differentiated from quotes from non-clinical sample |
| (Cassaniti et al., 2025) | The study aims to investigate how local Thai culture shapes the content and experience of hearing voices in schizophrenia. | Qualitative methodology is appropriate to explore cultural differences | Data were collected through interviews | Participants comprised 20 in-patients at Suan Prung Hospital who carried an official diagnosis of schizophrenia or paranoid schizophrenia. | Prim transcribed the Thai audio recordings of the interviews | NR | Institutional Review Boards of Stanford University (#19538), Washington State University (#17283), and Suan Prung Hospital (#60340) | This approach is sometimes described as “grounded theory,” | Themes are discussed with supporting quotes | The study is robust, meeting its objectives through a well-described sample |
| (Chernomas et al., 2000) | The research question for the needs assessment was: What are women’s perceptions of their experience with schizophrenia or schizoaffective disorder in the context of their life stages and corresponding health issues? | A qualitative methodology is appropriate to seek women's personal experiences with schizophrenia | Five different focus groups were organized for the aim of the research | A purposive sampling strategy was used to select 28 women | The sessions were audiotaped, and verbatim transcriptions were analysed for thematic content by the program coordinator and two researchers. | NR | NR | No specific references for thematic synthesis are reported | Findings are clearly organized into themes and supporting quotes are provided | Clinical and demographic characteristics of the sample contribute to the value of the research |
| (Clements et al., 2020) | This study aimed to explore how adults with lived experiences of voice hearing, who have participated in hearing voices  groups based on approaches of the Hearing Voices Movement, understand their voice hearing experiences (VHE). | A qualitative methodology is appropriate to explore patients lived experience with voice hearing | A phenomenological approach was  used to elicit voice hearers’ insights of their VHE to expand  the current understanding of this population within an Australian context. | Thirty-four potential participants were identified via a data  base search conducted by staff of the service. | Audio-recorded interviews lasting 70–120 min were  conducted in a private room at a community mental health  centre. | Study findings represent the researchers’ interpretations  of participants’ understanding of their experiences. Since  taking an objective stance is not possible, researchers must  be aware of, and reflect upon how personal biases may  influence the research process | Ethical approval was  obtained from the Northern Sydney Local Health District  Human Research Ethics Committee. | Thematic analysis was employed to  identify and explore the relationships between emerging  themes (Braun and Clarke 2006). | Findings are organized into themes and sub-themes in table 2 | Findings are clearly discussed, and sociodemographic characteristics of the sample are clearly reported |
| (Corin, 1998) | We wanted to examine to what extent certain types of transformation of the experience may be associated with better outcome | A qualitative methodology is appropriate to examine schizophrenic patients experiences | Open-ended interviews elicited four dimensions of relating to the world. | All were between 25 and 50 years old  and lived in the catchment area of the  Douglas Hospital, a lower middle and  working-class residential area. | Data were gathered through a series of three to six extensive, open-ended interviews conducted in a location chosen by the participant to ensure their comfort. | NR | NR | Concepts and methods developed by  European psychiatric phenomenology and by Ricoeur's hermeneutics are used  for discussing data collected in Montreal with patients diagnosed as schizophrenic. | Findings are divided into themes and supporting quotes are provided | The study successfully achieves its objective without apparent major flaws. |
| (De et al., 2013) | In an attempt to find out the frequency, criteria for classification, and other clinical aspects of bizarre delusions and  justification of their importance in the diagnostic system, this retrospective study was conducted. | A qualitative methodology is appropriate to justify the importance of diagnostic system | Records consisted of Diagnostic Interview for Genetic Studies (DIGS) interviews and available  medical records of DSM IV diagnosed SZ subjects  recruited in various research projects from 1996 to  2011. | subjects  recruited in various research projects from 1996 to  2011 | All subjects were interviewed using all the sections  of the DIGS. | NR | Ethical  permission was obtained from the Institutional Ethics  Committee. | The specific strategy for qualitative data analysis is not reported | Findings are grouped in categories | The large sample size adds significant value to the findings of the study. |
| (Dickson et al., 2016) | The aim of this study was to investigate participants' experiences of early interpersonal adversity and the way they link this with later experiences of PDs. | A qualitative methodology is appropriate to understand subjective experiences of adversities | A semi-structured interview schedule was developed in collaboration with two consultant clinical psychologists with ex tensive experience of working with people with PDs and psychosis and a researcher with an expertise in IPA. | Participants were recruited from two National Health Service (NHS) community mental health teams and one early intervention psychosis service in Northern England. | All interviews were recorded and transcribed verbatim, and participants allocated an alias name. | NR | NR | Analytic procedures followed IPA guidelines | Findings are clearly organized into themes and subthemes | Sample characteristics are reported, thus confirming the value of the research |
| (Fascendini et al., 2025) | The present study aims, first, to qualitatively investigate the basic forms of subjectivity underpinning primary delusions in individuals with schizophrenia through a phenomenological approach; second, to compare the experiential structure of these delusional phenomena with the experience of the sacred, as described in religious and anthropological accounts; and third, to fill the existing gap in the literature on this topic. | Qualitative methodology can be considered appropriate to explore subjectivity | Data were collected through in-person, semi-structured, and in-depth interviews. | All participants were enrolled from the Psychiatric Unit of the University Hospital of Parma. | Interviews were conducted in Italian and lasted between 35 min and 100 min, often split into two or more sessions. | NR | Local Institutional Ethics Committee of Area Vasta Emilia Nord (AVEN, Emilia-Romagna region, Italy; approval date: 4 April 2023, protocol code: 5761 – MYS_23). | Data analysis followed the seven steps of the IPA procedure | Findings are clearly reported with supporting quotes | The study met its objectives, used a well-described sample, discussed results thoroughly, and filled a specific gap in the literature |
| (Feyaerts et al., 2021) | This study aimed to investigate the nature of delusional reality experience, and its subjective apprehension,  in individuals with lived experience of delusions and a schizophrenia-spectrum diagnosis. | A qualitative methodology is appropriate for the purpose of this research | All participants gave written informed consent, including  for use of anonymised quotes. | We used purposive homogenous sampling to identify  participants for whom the research question was  important." | All interviews were audio  recorded and transcribed verbatim. | NR | Ethical  approval for the study was obtained from Ghent  University Hospital Ethics Committee (EC/2019/0042). | Data analysis followed the standard principles of IPA,  an idiographic phenomenological approach that aims  to understand how individuals make sense of their  experiences. | Themes and subthemes are reported clearly in a panel | The diversity of the sample helps strengthen the generalizability of the results. |
| (Fierro et al., 2018) | To explore the subjective experience of patients with delusion of control and to propose a psychopathological explanation based on empirical evidence. | A qualitative methodology is appropriate to explore subjective experiences | Qualitative exploratory study of 7 patients (6 with schizophrenia and 1 with schizophreniform disorder). A phenomenologically oriented semi-structured interview was used. | Seven patients were included (6 men and 1 woman). | All interviews were conducted entirely by the psychiatrist researcher and recorded on video. | NR | The protocol was approved by the university’s ethics committee on human research. | NR | Results for each case are described separately | The sample lack heterogeneity, however detailed data are reported, which adds value to the results obtained. |
| (Flanagan et al., 2012) | The study investigates the relationship between the personal experiences of schizophrenia and its depiction in the Diagnostic and Statistical Manual of Mental Disorders | A qualitative methodology is appropriate to investigate personal experiences | This qualitative method is  helpful for understanding in-depth a person’s  subjective experience, but it does not establish the representation of these experiences in  the population or the reliability and validity  of the measurement of these subjective experiences. | Participants in this study responded to  flyers posted at a mental health centre indicating the researchers’ interest in talking with people about their experiences of schizophrenia or schizoaffective disorder. Participants were recruited until saturation of themes was reached | All inter views were audiotaped and then transcribed  verbatim. | NR | This study was approved by the Hu man Investigation Committee of the Yale  School of Medicine. | The data were analysed using Interpretive Phenomenological Analysis | Qualitative data are grouped per symptom category | Sample characteristics contribute to generalize data |
| (Gee et al., 2019) | The aim of the current study was to investigate lived experiences of negative symptoms through secondary analysis of interviews conducted with individuals recovering from first-episode psychosis. | A qualitative methodology is appropriate to analyse interviews | Participants in the study's qualitative component (n = 207) were invited to be interviewed every 12 months during the 2 year follow-up period. | A purposive sample of transcripts of interviews with Super EDEN participants who participated in the qualitative sub-study described above was selected for the purposes of the current study. | The interviews that generated the data explored in this secondary analysis explored a range of topics, including symptoms, relationships, treatment and recovery. | NR | The study obtained NHS ethical approval before commencing and adhered to Good Clinical Practice guidelines. | A thematic approach to analysis was adopted. | Findings are clearly organized into themes | The sample is large and heterogeneous thus permitting data generalizability |
| (Gordon et al., 2023) | The  aim of this study was to provide a nuanced presentation of the impact of cultural context on  phenomenological experiences of paranoia. | A qualitative methodology is appropriate to explore the impact of cultural context | Archival interviews using the BPRS (N=21) from several published (17, 18) and  unpublished institutional review board–approved studies were transcribed and verified by  two authors | Participants were community-dwelling outpatients who had been enrolled  in one of three research studies conducted between 2017 and 2020 | Members of the research team transcribed available prerecorded (audio or video) BPRS  interviews from participants in previous research studies who had consented to interview  recording and for whom accompanying demographic information was available. | NR | unpublished institutional review board–approved studies were transcribed | Coding procedures for thematic content analysis were implemented to identify different  themes of paranoia across the sample | Emerging codes are reported | Clinical and demographic characteristics of the sample contribute to the value of the research |
| (Hansen et al., 2022) | The aim was to explore the potential applicability of meta motivation to schizophrenia  spectrum populations | A qualitative methodology is appropriate to explore meta motivation in schizophrenia | A semi-structured interview guide was developed by three research  team members (DL, MH & AM) and an expert consultant on qualitative  research (AS). | Participants in this study (N = 21) were recruited from a community  based recovery-oriented program for people with severe mental illness,  as part of a larger study on motivational enhancement in schizophrenia. | Semi-structured interviews were conducted in-person as part of  baseline assessments and transcribed by hand. | NR | Participation was completely  voluntary and ethical approval was given by the Institutional Review  Board. | A thematic  analysis approach was used and coding was completed by two re searchers. | Results are clearly divided into paragraphs | The study is valuable because it successfully meets its objective of introducing a novel and clinically relevant concept to its field. |
| (Hirschfeld et al., 2005) | The aim of this study was to explore and produce a representation of the subjective experiences of young men with psychosis and the meaning this has for them. | A qualitative methodology is appropriate to explore subjective experiences of young men with psychosis | This study used an in-depth qualitative method to explore young men’s subjective experiences of psychosis. | Participants were recruited to the study by sending information to young men via their keyworkers. | Auditing the transcript analyses enabled the thinking of the researcher to be followed and appraised | NR | The participants were recruited, with ethical approval from two mental health trusts. T | The qualitative analytic method of identifying initial descriptive codes emerging from the data was used | Findings are clearly summarized into emerging themes | Although the study included only male participants, the most relevant demographic characteristics are reported, adding value to the research. |
| (Isham et al., 2021) | We aimed to discover from patients: whether grandiose delusions have harmful consequences; the psychological mechanisms that maintain them; and what help patients may want from clinical services. | A qualitative methodology is appropriate for the aim, involving patients' experiences | A qualitative interview design was used to explore patients’ experiences of grandiose delusions. | Potential participants were identified by their clinical teams and, if consent was given to do so, approached by the lead author who provided information about the study and screened for suitability. | Semi-structured, in-depth, audio-recorded interviews were conducted by the lead author in accordance with relevant guidelines | Almostallparticipantswereunknowntotheinterviewer(aclinicalpsychologistinthe Trust) before the study. | The study was approved by the NHS Health Research Authority (REC reference: 17/SC/ 0515). | All interviews were coded by the lead author, however in line with recommendations (Barbour, 2001), multiple coding for a number of interviews, team reviews of the coding framework, and regular team consultation, including where uncertainty arose during coding, aimed to increase reliability. | Relevant findings are summarized in a panel | The sample differs for gender and age; therefore, data are considerate generalizable |
| (Jakes et al., 2004) | We wished to investigate whether the same motivational themes occur in the delusions and in the life problems/life goals of people with a psychosis. | A qualitative methodology is appropriate to explore motivational themes | We administered a semi-structured interview to all participants. We asked the participants to explain their delusional belief in their own words. We also asked them to describe the problems and goals they had at the present time and in the past. | Subjects were recruited consecutively from those referred. | The interviewer wrote down the response of the client verbatim and read the statement/s written down back to the participant to check that they agreed with what had been written down. | NR | NR | To derive a system of motives we employed a set of steps similar to those followed by Forgus and DeWolf (1974) | Few qualitative data are reported and no supporting quotes are present in the text | Only the qualitative findings from a single participant are reported and discussed; the lack of adequate thematic synthesis limits the value of the study. |
| (Jarosinski, 2008) | To understand hallucinations from an individual perspective of self. | A qualitative methodology is appropriate to understand the individual perspective | Heideggerian and hermeneutics-guided thematic analysis. Twelve individuals described their experiences of hallucinations. | Sampling was purposive. A heterogeneous sample was obtained by using two sites for recruitment in southeastern Virginia (Portsmouth and Richmond). | All interviews were recorded (with consent) and transcribed verbatim. | NR | Following approval by the institutional review board, participants gave written consent and provided their own pseudonyms. | Martin software (Diekelmann et al., 1995) was used to encode the data. | Supporting quotes are provided and findings are divided into themes | Sample included different genders and ages, thus permitting generalizability |
| (Johnson et al., 1997) | The purpose of this qualitative study was to increase the understanding of the experiences  of individuals with thought disorders, which precede incidents of aggression. | A qualitative methodology is appropriate to understand themes that precede aggressive behaviour in psychosis | This study used a qualitative, descriptive design in which  participants were invited to describe their experiences, pre  ceding aggression, in their own words, including whatever  details of context they chose to mention. | The sample of 11 men and one woman was selected  from two hospitals: a 289-bed tertiary care provincial psychiatric hospital and a general hospital with a 24-bed psychiatric unit, each of which was located in an urban area. | Data were collected through tape-recorded, individual  interviews. | NR | The study reports obtaining both written and verbal consent from participants and used pseudonyms to ensure confidentiality. | Analysis proceeded according to the method described by Giorgi (1985), which was adapted as an approach to content analysis | Findings are clearly organized into major themes | The characteristics of the sample and the broad discussion of themes give value to the research |
| (Jones et al., 2016) | To investigate the subjective experience of agency in the onset and early development of psychosis | A qualitative methodology is appropriate to explore subjective experiences | We conducted 19 in-depth interviews with a sample of individuals with self-reported diagnoses of schizophrenia and/or affective psychosis. | Interviews followed a semi-structured phenomenological protocol. | NR | During interviews, participants were intentionally made aware of the shared experience of the interviewer(s). For instance, interviewers explicitly acknowledged the difficulty involved in articulating unusual experiences, which often helped encourage farther-reaching attempts to grapple with the limitations of available vocabulary. | The project was approved by the primary investigator’s ethics board and all participants completed a standard consent process. | Our analytic strategy drew on both grounded theory (Charmaz, 2014) and qualitative phenomenology. | Findings are clearly divided into themes and supporting quotes are provided | The size and demographic characteristics of the sample make the research valuable |
| (Judge et al., 2008) | The purpose of this study was to examine the narrative processes by which individuals make sense of psychosis | A qualitative methodology is appropriate to explore narrative processes | A retrospective study investigating how individuals recognized and responded to early psychosis was carried out | Participants were 15 adults referred through clinicians in a public outpatient psychiatry clinic | Interviews were audiotaped and transcribed. | NR | Institutional re view board approval was obtained be fore data collection. | Analyses of interview transcripts were conducted by using inductive principles, including grounded theory and content analysis (9). | Findings are divided into main themes and supporting quotes are provided | Clinical and demographic characteristics of the sample contribute to the value of the research |
| (Knudson & Coyle, 2002) | The present  study attempts to explore in some depth the personal experience of two individuals  who hear voices | A qualitative methodology | Each participant was interviewed using a semi-structured interview schedule. | Potential participants for the study were contacted from the case-load of a consultant  psychiatrist working in the NHS in the south east of England. | Transcripts of the interviews | NR | NR | Transcripts of the interviews were subjected to interpretative phenomenological  analysis (IPA) | Findings are clearly reported and supporting quotes are provided | Even if 5 patients were interviewed, data from 2 participants only were reported |
| (Larsen, 2004) | The focus and rationale of the article is specified in a presentation of the cultural phenomenological approach to the study of experience and a discussion of some studies into the reflexivity of people suffering from mental illness. | A qualitative methodology is appropriate to study personal experience of people with mental illnesses | The arguments I present here arise from an ethnographic study I conducted of an experimental early intervention program in Copenhagen, Denmark. The program, called OPUS, included young people diagnosed for the first time within the “schizophrenic spectrum,” | The paper specifies that 15 key informants were selected from a larger cohort within a mental health program. However, it does not describe the specific recruitment strategy or the criteria used for selecting these particular participants, making it impossible to fully assess its appropriateness. | The data presented here are primarily drawn from the half-yearly individual interviews with my key informants, which were audio-recorded, transcribed verbatim, coded using broad empirical themes, and analysed using case and cross-case analytic strategies. | NR | NR | The computer software QSR NVivo facilitated data management. | Even if findings are deeply discussed, they are not clearly divided into themes | Overall, the study is valuable and robust. It uses an appropriate methodology with extensive data collection and rigorous analysis to offer significant and nuanced insights into the lived experience of psychosis. |
| (Löfs & Rasmussen, 2025) | This study examined the phenomenology of auditory verbal hallucinations (AVH) and visual hallucinations (VH) in schizophrenia and their relation to other psychopathology, particularly basic self-disorders. | Qualitative methodology is appropriate to explore the phenomenology of hallucinations | We included patients with a research diagnosis of schizophrenia-spectrum psychosis who participated in a larger psychopathological study focusing on subjective anomalies in schizophrenia-spectrum disorders. | The original study consecutively recruited patients from non-acute wards and outpatient facilities within the Mental Health Services of the Capital Region of Denmark. | Approximately 60% of participants consented to video recording of their interviews. After each interview, the interviewer prepared a detailed narrative summary covering all sections of the interview schedule. | NR | According to Danish legislation, approval from the Danish National Committee on Health Research Ethics (TDNCHRE) is not required for interview studies of this kind. | No analysis for qualitative data is provided in the main manuscript | Qualitative findings are mainly reported in supplementary materials | The study met its objectives, provided a thorough discussion of results, and offered significant clinical implications |
| (Marshall et al., 2020) | The aim of this study was to build a greater understanding of body image in individuals with persecutory delusions. Potential gender differences will be explored. | A qualitative methodology is appropriate to understand body image from the patients' perspective | This was an exploratory qualitative IPA study utilizing semi-structured interviews to explore body image in patients with persecutory delusions. | In line with IPA methodology, a purposive homogeneous sampling technique identified potential participants with experience of persecutory delusions, for whom the research question was meaningful (Smith, Flowers & Larkin, 2009). | Interviews were conducted by EM at participants’ homes or their local mental health clinic. Before the interview, participants were invited to choose a pseudonym for illustrative quotes. Interviews were audio-recorded and transcribed verbatim. | Therewerenoprior relationships between the researcher conducting the interviews and the participants. | The study had full ethical approval from the NHS Health Research Authority (HRA) (ref. 17/SC/0530). | Interpretative phenomenological analysis (Smith, 1996) was used to analyse participants’ accounts. | Findings are divided into paragraphs and reporting quotes are provided | The sample differs for gender and age; therefore, data are considerate generalizable |
| (Moernaut et al., 2018) | In this study, we make use of the Lacanian framework to explore the content of hallucinations. | A qualitative methodology is appropriate to explore the content of hallucinations | The interviews were semi-structured and guided by overall principles of qualitative interviewing | The participants in this study were recruited in the context of a bigger project concerning the experience of psychosis. | At first, the interviews were transcribed verbatim. | NR | The ethics committee of Ghent University Hospital approved this project. | To answer our first research question, we used thematic analysis, as outlined by and with support ofNVivo11software. | Findings are clearly summarized in a table | The study provides a detailed account of the clinical characteristics of the sample. |
| (Moernaut et al., 2022) | This paper aims at addressing this shortcoming by means of an  Interpretative Phenomenological Analysis of semi-structured interviews  with 12 participants with a psychotic disorder. | A qualitative methodology is appropriate to explore narratives | We conducted in-depth semi-structured interviews with fourteen participants | All  participants were currently treated for a psychotic disorder, as defined in DSM-5 (APA, 2013) under  the category of Schizophrenia Spectrum and Other Psychotic Disorders. | All interviews were audio recorded  and transcribed verbatim. The interviews and data analysis were conducted in Dutch. | During data collection, the interviewer (NM) visited both wards regularly to develop contacts with  possible participants. | The study obtained ethical  approval from the Ethics committee of the Ghent University Hospital. | Data analysis followed the principles of Interpretative Phenomenological Analysis (IPA; Smith &  Osborne, 2008) | Results are clearly divided into themes | The sample included different age and genders |
| (Møller & Husby, 2000) | Several questions present themselves for research: 1. Which prodromal changes in subjective experience are reported by first episode DSM-IV schizophrenia patients? 2. What promotes or restrains the subjects' perception, interpretation, and communication of these experiences? 3. Which prodromal changes in behaviour are reported by their families and friends (or even the patients them selves)? | A qualitative methodology is appropriate to answer the research questions proposed | we had to explore and describe naturalistic initial prodromes and experiences, as well as behaviour through open-ended, in-depth interviews | Retrospective interviews were performed as soon as diagnosis was established and overt psychosis resolved | Transcripts, records, reports, diaries, and notes were condensed according to our chosen conceptual framework and research questions | NR | The regional committee on ethics in medical research gave approval to this project. All participants gave written informed consent. | Phenomena were then tentatively grouped, taking into account (1) classifications proposed by the DSM-III-R and other reports; (2) the striking predominance of some phenomena in our data; and (3) the intuitive clinical judgment (face validity) by a large group of clinicians and clinical researchers independent of this project. | Findings are summarized in a table and supporting quotes are provided | The sample with mixed characteristics adds value to the study |
| (Ng et al., 2012) | The objective of this paper was to examine the coping strategies on auditory hallucinations, of Chinese people with schizophrenia in Hong Kong. | A qualitative methodology is appropriate to explore coping strategies | As the coping with the problem of hearing voices is subject to the hearers’ own interpretations, a qualitative research method therefore was adopted to facilitate the participants’ ability to describe the phenomena of the voices in fine detail and in their own terms. | The participants were recruited through non governmental organizations (NGOs) providing services for people with mental illness in Hong Kong. | With the consent of the participants, the interviews were digitally recorded. | NR | Invitation letters and a brief research proposal were sent to the directors of five NGOs for ethics review and approval. | Paragraph 4.3. Data analysis | Findings are clearly organized into themes and supporting quotes are provided | Demographic characteristics of the sample are reported, giving value to the findings |
| (Noroozi et al., 2025) | This study aimed to examine the lived experiences of autonomy among people who hear voices | Qualitative methodology is appropriate to explore lived experiences | In the present study, we adopted interpretative phenomenological analysis (IPA) to explore the lived experiences of autonomy among individuals who hear voices, focusing on the subjective meaning of autonomy from their perspective. | This study purposively recruited adults from a voices research participant registry | All interviews were audio-recorded and transcribed verbatim by the first author. | NR | Ethics approval was granted by the Swinburne University of Technology Human Research Ethics Committee (20215815-8163). | The analysis followed the data analysis process in IPA | Findings are clearly reported with supporting quotes | The study met its objective, used a well-described sample, and provided a thorough discussion |
| (Pietkiewicz et al., 2021) | This study analyses the experiences of possession in patients diagnosed with schizophrenia. | A qualitative methodology is appropriate to explore patients' experience | This study is part of a larger project examining phenomena and symptoms reported by people seeking religious rituals to liberate themselves from demonic influence.. | Participants in this study were two men and two women, all Roman Catholics, aged between 21 and 30 years, diagnosed with schizophrenia, and labelled by members of local religious communities or families as “possessed” due to their odd behaviour, auditory, and coenesthetic hallucinations. | All interviews were video-recorded and assessed by three healthcare professionals | NR | This project was held at the Research Centre for Trauma and Dissociation, financed by the National Science Center, and approved by the Ethical Review Board at the SWPS University of Social Sciences and Humanities in Poland. | All video recordings were transcribed verbatim and analysed, together with researchers’ notes, using qualitative data-analysis software– NVivo10. Consecutive steps recommended for IPA were employed in the study | Findings are clearly divided into themes | The study provides a valuable contribution to an under-researched area. |
| (Rhodes et al., 2016) | This study aimed to explore the experience of refugees diagnosed with psychosis from a first-person perceptive. | A qualitative methodology is appropriate to explore subjective experiences | The interview schedule was based on questions proposed by the authors and other clinicians experienced in working in the area and the relevant literature | Seven refugees from a routine clinical service who had experienced trauma were interviewed. | Data were gathered using in-depth, semi-structured interviews | NR | Ethical approval was granted by the local research ethics committee of the relevant London NHS Mental Health Trust. | The analysis involves several processes and includes: repeated reading, making notes and observations on significant content, the generation of themes and looking for connections and patterns between themes until a coherent set is developed. | Findings are clearly divided into themes and supporting quotes are provided | Heterogeneity of the sample makes the research valuable |
| (Rhodes & Jakes, 2004) | we wished to examine what  participants said when attempting to justify the perceived truth of their beliefs and addressed two specific questions. | A qualitative methodology is appropriate to explore patients' experiences | Both therapists followed an agreed therapy procedure in that at a certain point in the work we would ask for ‘evidence’ for beliefs. | The participants, aged 18–65, all lived in the community, either independently or in hostels, and were referred by their  psychiatrists. | The authors first read the notes taken during the session: these were then written up in the form of detailed summaries, one for each participant. | The first author saw 11 participants for therapy, and the second saw 12. | NR | The next step involved the generation of thematic codes: here we carried out a thematic analysis drawing on the procedure described by Smith (1995) and Smith, Jarman, and Osborne (1999). | Findings are grouped for category and supporting quotes are provided | The study is valuable due to its clear aims, appropriate methodology, and particularly rigorous data analysis that included inter-rater reliability checks. |
| (Rhodes & Healey, 2017) | The study aimed to investigate the experience of adults with a diagnosis of psychosis and who have survived childhood physical abuse. | A qualitative methodology is appropriate to explore childhood abuse | A semi-structured interview schedule was used to guide the interview, which contained questions and prompts to elicit information on participants’ current problems and difficulties experienced across the lifespan. | participants were recruited from a hospital based psychology department. | A semi-structured interview schedule was used to guide the interview | NR | approval from the appropriate local Ethics Committees | The procedure used to analyse the interview transcripts broadly followed the stages described in Smith and Osborn (2003). | Findings are clearly divided into themes and supporting quotes are provided | Heterogeneity of the sample makes the research valuable |
| (Rieben et al., 2013) | The aim of the present study was therefore to elicit how patients with delusions with religious contents conceptualized or experienced their spirituality and religiousness, using an in-depth analysis of the patients’ interviews. | A qualitative methodology is appropriate to explore patients' spirituality | Spirituality, religiousness, and spiritual/ religious coping were elicited through a semi structured interview. | Two hundred thirty-six outpatients were included, 115 patients were randomly selected from Geneva’s four public ambulatory psychiatric facilities, and 121 consecutive outpatients were recruited from the Assertive Community Treatment program in Trois-Rivières. | Interviews were audiotaped and transcribed verbatim. | NR | This study was approved by the ethical committees of the University Hospital of Geneva and the Trois-Rivières University. | Therefore, a third clinician (I. R.) analysed these 62 interviews using the grounded theory’s coding steps to create an exploratory frame work that would provide a phenomenological description of the patients’ religious delusions and their relationships with spirituality and religiousness. | Findings are clearly organized into themes and deeply discussed | Sample size large dimension, unusual for qualitative research, make the findings valuable |
| (Shepherd et al., 2012) | The aim of this study was to gain an understanding of the perspectives of older adults with schizophrenia about if and how their experience of the illness had changed over the lifespan as well as their expectations for the future. | A qualitative methodology is appropriate to understand the perspective of older adults with schizophrenia | Based on consensus meetings, a semi structured interview format was developed | Participants were recruited by informing other Center investigators of the study and through dissemination of study information via Center recruitment staff. | Interviews were audiotaped and transcribed | NR | This study was approved by the UCSD Institutional Re view Board, and a written informed consent was obtained from each participant. | Audiotapes were transcribed and were processed using the grounded theory-based approach of ‘‘coding consensus, co-occurrence, and comparison’’. | Findings are clearly summarized | Sample size and demographic characteristics of the sample contribute to the value of the research |
| (Simmons et al., 2024) | We sought to better understand the burden of illness associated with schizophrenia in patients’ own words, as well as their experiences with previous and current treatments and how they have impacted their daily life. | A qualitative methodology is considerate appropriate for the aim of the study | Semi-structured exit interviews are appropriate to a qualitative methodology | Interviews were conducted by trained qualitative researchers who had experience working with individuals with mental illness. All interviews lasted approximately 60 minutes. | "All interviews were audio-recorded and transcribed." | NR | NCT02873208) | NVivo, version 11.0 | Emerging themes are clearly divided into paragraphs | The research involves participants of different genders and racial/ethnic backgrounds, making it reasonably generalizable. |
| (Skourteli et al., 2025) | The aim of the present study is to investigate the subjective, lived experiences of service users and their families regarding the onset of psychosis and the pathways in seeking appropriate treatment as well as the barriers they may have encountered during this process. | Qualitative methodology is appropriate to explore lived experiences | Six in-depth individual interviews were conducted (4 service users and 2 mothers). | Recruitment was purposeful following the snowball sampling technique. | All interviews were conducted in the premises of the Day Centre and were audio recorded and transcribed verbatim. | The interviews were conducted by the first two authors, with each interview lasting approximately 90 min. The first author was an experienced psychoanalyst and researcher, independent of the Day Centre and the second was an experienced psychologist and member of the broader multidisciplinary team of the Day Centre. | The present study was approved by EPAPSY’s Ethics Committee and took place with the informed consent of all participants. | We used thematic analysis with an experiential and realist orientation (Braun & Clarke, 2006) to analyses the data derived from the narratives that emerged within individual interviews. | Findings are clearly reported with supporting quotes | Despite a small sample size, the study met its objectives |
| (Startup et al., 2015) | The aim was to find out what patients with persecutory delusions report are the factors that bring a worry episode to an end. | A qualitative methodology is appropriate to explore delusions from individual perspective | An open-ended semi-structured interview technique and IPA qualitative analysis was employed | AllparticipantshadbeenrecruitedfrommentalhealthteamsacrossOxfordHealthNHS Foundation Trust and Southern Health NHS Foundation Trust. | Participants were interviewed in a private room within their home or at their local mental health team base. The interviews were recorded on a digital Dictaphone and transcribed verbatim into written text. | NR | Approval for this study was gained through the local research ethics committee (ref number: 11/SC/0001). | The transcripts were qualitatively analysed using IPA as described by Smith and Osborn (2008). | Findings are clearly reported with supporting quotes | The study provides a detailed account of the clinical characteristics of the sample. |
| (Stephane, 2024) | (1) Experience of double reality (2) Emergence and development of two realities (3) Truth quality of psychotic or private reality (4) Insight into illness (5) Communication of psychotic experiences | A qualitative methodology is appropriate to explore the experience of double reality in schizophrenia patients | The interviews were semi structured and conversational giving the patients ample possibility to describe their experiences. | The patients were recruited from psychiatric services of the Capital Region of Denmark: Psychiatric Center Glostrup, Psychiatric Center Copenhagen, and Psychiatric Center Amager. All these services are affiliated with University of Copenhagen. | All interviews were audiotaped and subsequently transcribed. | NR | The patients participated on the condition of informed,  written consent and the study was approved by the Data  Protection Agency (P-2020-4), University of Copenhagen  (514-0045/19-4000), and the ethics committee of University  of Copenhagen. | The data analysis consisted in obtaining a consensus about the four target domains (top-down approach) following the principles of qualitative, thematic analysis. | Findings are clearly organized into themes and supporting quotes are provided | Given the characteristics of the sample, the data can be considered generalizable |
| (Stopa et al., 2013) | This research examined the experience of being under threat in social phobia and paranoia. | A qualitative methodology is appropriate to explore threat experiences | The interview consists of a standard set of questions and prompts. The semi-structured format allows participants to expand on questions if relevant. Interviews lasted between 20 and 60 minutes, and were recorded with participants’ permission. | Participants with social phobia and persecutory delusions were recruited to this research, andalinkedstudycomparingthesegroupswithclinicalandnon-clinicalcontrols | The semi-structured interview was completed (along with questionnaires for the linked study) and audio-recorded for later analysis. | NR | NR | The transcripts were analysed following thematic methods | Supporting quotes are provided and diagnosis are specified | The inclusion of diagnosis other than psychosis gives value to the research |
| (Taylor et al., 2020) | This study utilised a qualitative approach to explore core schematic beliefs in psychosis  (strongly held positive and negative beliefs about the self and others) and their relation to  hallucinations and delusions. | A qualitative methodology is appropriate to explore core beliefs in psychosis | The study used an exploratory qualitative design. | Study recruitment continued until data saturation was reached | The individual interviews were digitally recorded and transcribed verbatim. | There was no previous relationship with any interview participants. | The study received ethical approval from the National Health Service (NHS) Health  Research Authority (NHS HRA), UK (13/NW/0264) and permission to proceed from  Lancashire Care NHS Foundation Trust and Greater Manchester West Mental Health NHS  Foundation Trust (Refs: 124272 and 782) in the North West of England. | The interview  data was analysed using a thematic analysis approach, using a six-phase approach. | Findings are clearly divided into major themes and supporting quotes are provided | Clinical and demographic characteristics of the sample are clearly reported |
| (Toh et al., 2020) | The current study aimed to address gaps in existing knowledge by: a) comprehensively describing AVH phenomenology by diagnosis and mood state in a large-scale affective and non-affective psychosis sample with the use of well-validated clinical measures, b) investigating significant predictors of voice-related distress and functional impairment based on machine-learning algorithms, and c) conducting qualitative thematic analysis of participants’ AVH descriptions encompassing rich, subjective experiences (as well as uncover facets overlooked by quantitative research) | A qualitative methodology is appropriate to address phenomenological questions | Phenomenological assessments of AVHs comprised in-depth, semi-structured interviews based on amalgamation of conventional psychosis measures– | Participants were recruited from: a) public and private mental health services in Melbourne, Australia, b) community venues, or c) online advertising on research participation forums. | Participants’ responses to an open-ended question: “Could you describe your experiences of hearing voices and other sounds, providing examples where you feel comfortable?” were written down verbatim, de-identified and collated for further analysis. | NR | The study was approved by institutional human research ethics committees at the Alfred Hospital, St. Vincent's Hospital and The Melbourne Clinic. | To address the third aim involving qualitative synthesis, our data underwent thematic analysis (Braun and Clarke, 2006) via an inductive (data-driven) iterative process led by first author WLT (an academic psychologist). | Qualitative findings are summarized in a figure | The sample is heterogeneous, thus allowing the results to be generalized. |
| (Toh et al., 2024) | The current study therefore had three main aims: (i) a quantitative description of the characteristics of nonauditory hallucinations in a transdiagnostic cohort, (ii) groupwise comparisons of these phenomena in nonaffective versus affective psychosis, and (iii) a qualitative thematic analysis based on patients’ descriptions of their nonauditory hallucinations | A qualitative methodology is appropriate to address phenomenological questions | This study represented secondary analysis of a pre-collected dataset using a transdiagnostic group of psychosis patients experiencing primary auditory hallucinations. | Participants were recruited from public and private mental health services, community venues, or online advertising on research participation forums and were asked to attend a face-to-face session in Melbourne | Participant responses were recorded verbatim, de-identified, and coded for further analysis. | One of two researchers (an academic psychologist and a provisional psychologist), with no existing professional relationship with participants, conducted the interview. | The study was approved by institutional human research ethics committees at the Alfred Hospital, St. Vincent's Hospital and The Melbourne Clinic. | a phenomenological qualitative approach  was adopted, using thematic analysis (Braun et al., 2006) | Qualitative findings are summarized in figure 1 | The sample is heterogeneous, thus allowing the results to be generalized. |
| (Topor & Di Girolamo, 2010) | The aim of this article is to investigate how users with a psychosis diagnosis describe how they make choices to manage crises and to examine the consequences of their experiences for our understanding of psychosis and for clinical practice. | A qualitative methodology is appropriate to investigate how psychotic patients manage crises | This study is based on the transcripts from the 10 focus group meetings. | The members of a user organization, ‘‘Les Amis de l’Atelier du Non-Faire’’ Paris, France took the initiative for the study which was organized as a collaborative project. | The conversations in the focus groups were tape-recorded, transcribed and then presented to the participants before the material was analysed by the researchers. | NR | NR | They were analysed using a method based on Grounded Theory | Findings are divided into paragraphs and supporting quotes are provided | While the study's collaborative design is a strength, the lack of information regarding formal ethics committee approval raises a significant concern about procedural oversight. Additionally, the researchers acknowledge that their inability to select for maximal diversity or to analyses each session before the next could introduce bias and limit the generalizability of the findings. |
| (Upthegrove et al., 2016) | To conduct primary research into the nature of subjective experience of AVH in first-episode psychosis. | A qualitative methodology is appropriate to explore subjective experiences | Aphenomenologicalstudyusingdiaryandphoto-elicitation qualitative techniques investigating the subjective experienceofAVHin25youngpeoplewithfirst-episode psychosis. | Participants were recruited from the Birmingham Early Intervention Service, which covers areas of a diverse range of socio economic and ethnic communities | All dialogue was digitally recorded and transcribed verbatim. | NR | National Health Service ethical approval was obtained and included the anonymous publication of direct quotes (NRES 13/WM/0277) | Data were analysed using conventional content analysis. | Findings are clearly organized into themes and subthemes | Sample size, clinical and demographic characteristics of the sample make the research valuable |
| (Wahyuni et al., 2021) | This research is phenomenological and aims for describing the experiences of patients with schizophrenia who  had hallucination. | A qualitative methodology is appropriate to describe patients' experiences | This research is phenomenological and  aims for describing the experiences of patients with  schizophrenia who had a hallucination. | This research  got 15 patients involved in it. Inclusion criteria of my  study are patients with schizophrenia paranoid who  had hallucination. | NR | NR | This research has  been through an ethical clearance from the ethics  commission, Faculty of Nursing Universitas Sumatera  Utara. | The data analysis was Colaizzi’s method. | Findings are grouped into themes | The sample is heterogeneous, thus allowing the results to be generalized. |
| (Wood et al., 2015) | This study aims to enrich understanding of cognitive difficulties in psychosis, through gathering information about subjective experiences. | A qualitative methodology is appropriate to understand subjective experiences | Semi-structured interview questions were guided by the study’s aims (Smith et al., 2009) | Eleven participants were purposively recruited from mental health teams in a suburban NHS Trust | Interviews lasted 30–60mins and were recorded, transcribed and anonymized for analysis. | The researcher kept a research diary to facilitate bracketing of prior assumptions (e.g., in relation to experience delivering CRT) and minimize their influence on data gathering and interpretation | Ethical approval was obtained from a National Health Service (NHS) Research and Ethics Committee. | Interpretative phenomenological analysis (IPA) (Smith et al., 2009) was used | Findings are clearly summarized | Demographic and assessment information aided sample contextualization (Elliott et al., 1999) |
| (Xanthopoulou et al., 2022) | In this paper, we explored subjective accounts of people with psychosis, their family caregivers and mental health  practitioners involved in care delivery in NHS mental health  services, on social isolation and barriers/enablers to having  social contacts and feeling part of a community. | A qualitative methodology is appropriate to explore perspectives of patients and caregivers | Focus groups were conducted at each of the three participating Trusts, with each participant group: people with psychosis, carers and MHPs. | Participants were people with psychosis (PwP), family caregivers and mental health practitioners (MHPs), and were  identified through secondary care/community mental health  services. Carers also self-referred from posters displayed in  various outpatient community locations and in participating  Trust premises. | The focus groups  and interviews were audio recorded, transcribed, line-by-line  coded using the software program NVivo 12 and analysed  using inductive thematic analysis (Terry et al., 2017). | NR | Ethical approval was granted by the East of England Cambridgeshire  and Hertfordshire Research Ethics Committee (17/EE/0276). | Data were coded using the software program NVivo 12 and analysed  using inductive thematic analysis (Terry et al., 2017). | Results are organized into main themes | The sample included different ages and genders |
| (Yttri et al., 2020) | 1. The age of onset and duration of hallucinations before their disclosure and the naming of pathological experiences as being “voices.” 2. The patients' reaction to and their comprehension of these phenomena. 3. Reasons and circumstances for disclosure. 4. Associated experiences and the overall sense of subjectivity. 5. Insight in the pathological status of AVH. | Except for the first point, qualitative methodology is appropriate for the other aims of the research | Specifically, for this study, we have prepared a semi structured questionnaire consisting of several domains of interest. The questionnaire was constructed in the process of pilot clinical interviews, discussions within the research group, and studies of the literature. | Twenty patients with AVH and fulfilling the ICD-10 criteria of schizophrenia were included in the study. | All interviews were audiotaped and transcribed verbatim for the use of consensus discussions between the investigators to divide the transcripts into the target domains of the study. | NR | The ethical committee approved the study, and the patients participated on the condition of informed consent. | Details about data analysis are missing | Findings are clearly reported with supporting quotes | The sample differs for gender and age; therefore, data are considerate generalizable |

**Appendix E: ENTREQ checklist**

| **N.** | **Item** | **Guide and description** | **Page** |
| --- | --- | --- | --- |
| 1 | Aim | State the research question the synthesis addresses. | 1 |
| 2 | Synthesis methodology | Identify the synthesis methodology or theoretical framework which underpins the synthesis, and describe the rationale for choice of methodology (e.g. meta-ethnography, thematic synthesis, critical interpretive synthesis, grounded theory synthesis, realist synthesis, meta-aggregation, meta-study, framework synthesis). | 3 |
| 3 | Approach to searching | Indicate whether the search was pre-planned (comprehensive search strategies to seek all available studies) or iterative (to seek all available concepts until they theoretical saturation is achieved). | 2 |
| 4 | Inclusion criteria | Specify the inclusion/exclusion criteria (e.g. in terms of population, language, year limits, type of publication, study type) | 2 |
| 5 | Data sources | Describe the information sources used (e.g. electronic databases (MEDLINE, EMBASE, CINAHL, psycINFO, Econlit), grey literature databases (digital thesis, policy reports), relevant organisational websites, experts, information specialists, generic web searches (Google Scholar) hand searching, reference lists) and when the searches conducted; provide the rationale for using the data sources. | 2 |
| 6 | Electronic Search strategy | Describe the literature search (e.g. provide electronic search strategies with population terms, clinical or health topic terms, experiential or social phenomena related terms, filters for qualitative research, and search limits). | 2, Appendix A |
| 7 | Study screening methods | Describe the process of study screening and sifting (e.g. title, abstract and full text review, number of independent reviewers who screened studies). | 3, 4 |
| 8 | Study characteristics | Present the characteristics of the included studies (e.g. year of publication, country, population, number of participants, data collection, methodology, analysis, research questions). | 5 |
| 9 | Study selection results | Identify the number of studies screened and provide reasons for study exclusion (e,g, for comprehensive searching, provide numbers of studies screened and reasons for exclusion indicated in a figure/flowchart; for iterative searching describe reasons for study exclusion and inclusion based on modifications t the research question and/or contribution to theory development). | 3, Appendix B |
| 10 | Rationale for appraisal | Describe the rationale and approach used to appraise the included studies or selected findings (e.g. assessment of conduct (validity and robustness), assessment of reporting (transparency), assessment of content and utility of the findings). | 2 |
| 11 | Appraisal items | State the tools, frameworks and criteria used to appraise the studies or selected findings (e.g. Existing tools: CASP, QARI, COREQ, Mays and Pope; reviewer developed tools; describe the domains assessed: research team, study design, data analysis and interpretations, reporting). | 2 |
| 12 | Appraisal process | Indicate whether the appraisal was conducted independently by more than one reviewer and if consensus was required. | 2 |
| 13 | Appraisal results | Present results of the quality assessment and indicate which articles, if any, were weighted/excluded based on the assessment and give the rationale. | 20, Appendix D |
| 14 | Data extraction | Indicate which sections of the primary studies were analysed and how were the data extracted from the primary studies? (e.g. all text under the headings “results /conclusions” were extracted electronically and entered into a computer software). | 2, 3 |
| 15 | Software | State the computer software used, if any. | 3 |
| 16 | Number of reviewers | Identify who was involved in coding and analysis. | 3 |
| 17 | Coding | Describe the process for coding of data (e.g. line by line coding to search for concepts). | 3 |
| 18 | Study comparison | Describe how were comparisons made within and across studies (e.g. subsequent studies were coded into pre-existing concepts, and new concepts were created when deemed necessary). | 3 |
| 19 | Derivation of themes | Explain whether the process of deriving the themes or constructs was inductive or deductive. | 3 |
| 20 | Quotations | Provide quotations from the primary studies to illustrate themes/constructs, and identify whether the quotations were participant quotations of the author’s interpretation. | 10-11, 12-17, Appendix C |
| 21 | Synthesis output | Present rich, compelling and useful results that go beyond a summary of the primary studies (e.g. new interpretation, models of evidence, conceptual models, analytical framework, development of a new theory or construct) | 18-20 |

**References**

Alderson-Day, B., Woods, A., Moseley, P., Common, S., Deamer, F., Dodgson, G., & Fernyhough, C. (2021). Voice-Hearing and Personification: Characterizing Social Qualities of Auditory Verbal Hallucinations in Early Psychosis. *Schizophrenia Bulletin*, *47*(1), 228–236. https://doi.org/10.1093/schbul/sbaa095

Boydell, K. M., Gladstone, B. M., & Volpe, T. (2003). Interpreting narratives of motivation and schizophrenia: A biopsychosocial understanding. *Psychiatric Rehabilitation Journal*, *26*(4), 422–426. https://doi.org/10.2975/26.2003.422.426

Butcher, I., Berry, K., & Haddock, G. (2020). Understanding individuals’ subjective experiences of negative symptoms of schizophrenia: A qualitative study. *British Journal of Clinical Psychology*, *59*(3), 319–334. Scopus. https://doi.org/10.1111/bjc.12248

Campbell, M. L. C., & Morrison, A. P. (2007). The subjective experince of paranoia: Comparing the experiences of patients with psychosis and individuals with no psychiatric history. *Clinical Psychology and Psychotherapy*, *14*(1), 63–77. Scopus. https://doi.org/10.1002/cpp.510

Cassaniti, J., Sakulsriprasert, C., & Luhrmann, T. M. (2025). Wildness, Mindfulness, and the Phenomenology of Voice-Hearing in Thailand. *Schizophrenia Bulletin*, *51*(Supplement_3), S253–S260. https://doi.org/10.1093/schbul/sbaf137

Chernomas, W. M., Clarke, D. E., & Chisholm, F. A. (2000). Perspectives of women living with schizophrenia. *Psychiatric Services (Washington, D.C.)*, *51*(12), 1517–1521. https://doi.org/10.1176/appi.ps.51.12.1517

Clements, S., Coniglio, F., & Mackenzie, L. (2020). ‘I’m Not Telling an Illness Story. I’m Telling a Story of Opportunity’: Making Sense of Voice Hearing Experiences. *Community Mental Health Journal*, *56*(2), 196–205. https://doi.org/10.1007/s10597-019-00465-x

Corin, E. (1998). The thickness of being: Intentional worlds, strategies of identity, and experience among schizophrenics. *Psychiatry*, *61*(2), 133–146. https://doi.org/10.1080/00332747.1998.11024824

De, S., Bhatia, T., Thomas, P., Chakraborty, S., Prasad, S., Nagpal, R., Nimgaonkar, V. L., & Deshpande, S. N. (2013). Bizarre delusions: A qualitative study on Indian schizophrenia patients. *Indian Journal of Psychological Medicine*, *35*(3), 268–272. Scopus. https://doi.org/10.4103/0253-7176.119484

Dickson, J. M., Barsky, J., Kinderman, P., King, D., & Taylor, P. J. (2016). Early relationships and paranoia: Qualitative investigation of childhood experiences associated with the development of persecutory delusions. *Psychiatry Research*, *238*, 40–45. Scopus. https://doi.org/10.1016/j.psychres.2016.02.006

Fascendini, N., Amorosi, S., Dell’Eva, A., Marchesi, C., & Tonna, M. (2025). Primary delusional experiences in schizophrenia and the sacred: A qualitative study. *Philosophical Psychology*, 1–22. https://doi.org/10.1080/09515089.2025.2509795

Feyaerts, J., Kusters, W., Van Duppen, Z., Vanheule, S., Myin-Germeys, I., & Sass, L. (2021). Uncovering the realities of delusional experience in schizophrenia: A qualitative phenomenological study in Belgium. *The Lancet Psychiatry*, *8*(9), 784–796. Scopus. https://doi.org/10.1016/S2215-0366(21)00196-6

Fierro, M., Hernández, A. M., & Malcolm, J. (2018). The Psychopathology of Delusion of Control According to Subjective Experience. *Revista Colombiana de Psiquiatria*, *47*(4), 221–228. Scopus. https://doi.org/10.1016/j.rcp.2017.02.004

Flanagan, E. H., Solomon, L. A., Johnson, A., Ridgway, P., Strauss, J. S., & Davidson, L. (2012). Considering DSM-5: The personal experience of schizophrenia in relation to the DSM-IV-TR criteria. *Psychiatry*, *75*(4), 375–386. https://doi.org/10.1521/psyc.2012.75.4.375

Gee, B., Hodgekins, J., Lavis, A., Notley, C., Birchwood, M., Everard, L., Freemantle, N., Jones, P. B., Singh, S. P., Amos, T., Marshall, M., Sharma, V., Smith, J., & Fowler, D. (2019). Lived experiences of negative symptoms in first-episode psychosis: A qualitative secondary analysis. *Early Intervention in Psychiatry*, *13*(4), 773–779. Scopus. https://doi.org/10.1111/eip.12558

Gordon, S., Mote, J., & Fulford, D. (2023). Qualitative Analysis of Paranoia Reported in Clinical Interviews With Black and White Adults With Schizophrenia. *Psychiatric Services (Washington, D.C.)*, *74*(12), 1263–1269. https://doi.org/10.1176/appi.ps.20220089

Hansen, M. C., Lynch, D. A., Stefancic, A., & Medalia, A. (2022). Metamotivation in people diagnosed with schizophrenia: A conceptual introduction and qualitative study. *Schizophrenia Research*, *243*, 317–321. Scopus. https://doi.org/10.1016/j.schres.2021.06.003

Hirschfeld, R., Smith, J., Trower, P., & Griffin, C. (2005). What do psychotic experiences mean for young men? A qualitative investigation. *Psychology and Psychotherapy*, *78*(Pt 2), 249–270. https://doi.org/10.1348/147608305X25865

Isham, L., Griffith, L., Boylan, A.-M., Hicks, A., Wilson, N., Byrne, R., Sheaves, B., Bentall, R. P., & Freeman, D. (2021). Understanding, treating, and renaming grandiose delusions: A qualitative study. *Psychology and Psychotherapy: Theory, Research and Practice*, *94*(1), 119–140. Scopus. https://doi.org/10.1111/papt.12260

Jakes, S., Rhodes, J., & Issa, S. (2004). Are the themes of delusional beliefs related to the themes of life-problems and goals? *Journal of Mental Health*, *13*(6), 611–619. Scopus. https://doi.org/10.1080/09638230400024877

Jarosinski, J. M. (2008). Exploring the experience of hallucinations from a perspective of self: Surviving and persevering. *Journal of the American Psychiatric Nurses Association*, *14*(5), 353–362. Scopus. https://doi.org/10.1177/1078390308325417

Johnson, B., Martin, M.-L., Guha, M., & Montgomery, P. (1997). The experience of thought-disordered individuals preceding an aggressive incident. *Journal of Psychiatric and Mental Health Nursing*, *4*(3), 213–220. Scopus. https://doi.org/10.1046/j.1365-2850.1997.00041.x

Jones, N., Kelly, T., & Shattell, M. (2016). *God in the brain: Experiencing psychosis in the postsecular United States*. *53*, 488–505. Scopus. https://doi.org/10.1177/1363461516660902

Judge, A. M., Estroff, S. E., Perkins, D. O., & Penn, D. L. (2008). Recognizing and responding to early psychosis: A qualitative analysis of individual narratives. *Psychiatric Services (Washington, D.C.)*, *59*(1), 96–99. https://doi.org/10.1176/ps.2008.59.1.96

Knudson, B., & Coyle, A. (2002). The experience of hearing voices: An interpretative phenomenological analysis. *Published Journal Articles and Book Chapters from the Department of Psychology*, *13*.

Larsen, J. A. (2004). Finding meaning in first episode psychosis: Experience, agency, and the cultural repertoire. *Medical Anthropology Quarterly*, *18*(4), 447–471. Scopus. https://doi.org/10.1525/maq.2004.18.4.447

Löfs, B. M., & Rasmussen, A. R. (2025). Visual and Auditory Verbal Hallucinations in Schizophrenia and Their Relation to Self-Disorders. *Psychopathology*, 1–11. https://doi.org/10.1159/000548797

Marshall, E., Freeman, D., & Waite, F. (2020). The experience of body image concerns in patients with persecutory delusions: ‘People don’t want to sit next to me’. *Psychology and Psychotherapy*, *93*(3), 639–655. https://doi.org/10.1111/papt.12246

Moernaut, N., Krivzov, J., Lizon, M., Feyaerts, J., & Vanheule, S. (2022). Negative symptoms in psychosis: Failure and construction of narratives. *Psychosis*, *14*(3), 227–236. Scopus. https://doi.org/10.1080/17522439.2021.1948092

Moernaut, N., Vanheule, S., & Feyaerts, J. (2018). Content Matters, a Qualitative Analysis of Verbal Hallucinations. *Frontiers in Psychology*, *9*, 1958. https://doi.org/10.3389/fpsyg.2018.01958

Møller, P., & Husby, R. (2000). The initial prodrome in schizophrenia: Searching for naturalistic core dimensions of experience and behavior. *Schizophrenia Bulletin*, *26*(1), 217–232. https://doi.org/10.1093/oxfordjournals.schbul.a033442

Ng, P., Chun, R. W. K., & Tsun, A. (2012). Recovering from hallucinations: A qualitative study of coping with voices hearing of people with schizophrenia in Hong Kong. *The Scientific World Journal*, *2012*. Scopus. https://doi.org/10.1100/2012/232619

Noroozi, S., Doroud, N., Longden, E., & Thomas, N. (2025). Hearing voices and the quest for autonomy: An interpretative phenomenological analysis. *Psychology and Psychotherapy*. https://doi.org/10.1111/papt.70026

Pietkiewicz, I. J., Kłosińska, U., & Tomalski, R. (2021). Delusions of Possession and Religious Coping in Schizophrenia: A Qualitative Study of Four Cases. *Frontiers in Psychology*, *12*. Scopus. https://doi.org/10.3389/fpsyg.2021.628925

Rhodes, J. E., & Healey, L. J. (2017). ‘Many die in the hurricane’: An Interpretative Phenomenological Analysis of Adults with Psychosis and a History of Childhood Physical Abuse. *Clinical Psychology and Psychotherapy*, *24*(3), 737–746. Scopus. https://doi.org/10.1002/cpp.2043

Rhodes, J. e., & Jakes, S. (2004). Evidence given for delusions during cognitive behaviour therapy. *Clinical Psychology & Psychotherapy*, *11*(3), 207–218. https://doi.org/10.1002/cpp.417

Rhodes, J. E., Parrett, N. S., & Mason, O. J. (2016). A qualitative study of refugees with psychotic symptoms. *Psychosis*, *8*(1), 1–11. Scopus. https://doi.org/10.1080/17522439.2015.1045547

Rieben, I., Mohr, S., Borras, L., Gillieron, C., Brandt, P.-Y., Perroud, N., & Huguelet, P. (2013). A thematic analysis of delusion with religious contents in schizophrenia: Open, closed, and mixed dynamics. *Journal of Nervous and Mental Disease*, *201*(8), 665–673. Scopus. https://doi.org/10.1097/NMD.0b013e31829c5073

Shepherd, S., Depp, C. A., Harris, G., Halpain, M., Palinkas, L. A., & Jeste, D. V. (2012). Perspectives on schizophrenia over the lifespan: A qualitative study. *Schizophrenia Bulletin*, *38*(2), 295–303. Scopus. https://doi.org/10.1093/schbul/sbq075

Simmons, A., O’Sullivan, A. K., Carpenter-Conlin, J., Carty, M. K., Saucier, C., & McDonnell, D. (2024). Using qualitative exit interviews to explore schizophrenia burden and treatment experience in clinical trial patients. *Frontiers in Psychiatry*, *15*. Scopus. https://doi.org/10.3389/fpsyt.2024.1377174

Skourteli, M., Tritari, M., Mavreas, V., Konstantakopoulos, G., & Stylianidis, S. (2025). Duration of Untreated Psychosis: A Thematic Analysis of service-users’ subjective experiences of diagnosis and seeking treatment. *Trends in Psychology*. https://doi.org/10.1007/s43076-025-00467-7

Startup, H., Pugh, K., Cordwell, J., Kingdon, D., & Freeman, D. (2015). How do individuals with persecutory delusions bring worry to a close? An interpretive phenomenological analysis. *Behavioural and Cognitive Psychotherapy*, *43*(4), 465–477. https://doi.org/10.1017/S1352465813001136

Stephane, M. (2024). Hallucinations: Neurobiology and patient experience. *Schizophrenia Research*, *265*, 1–3. https://doi.org/10.1016/j.schres.2023.12.018

Stephensen, H., Urfer-Parnas, A., & Parnas, J. (2024). Double bookkeeping in schizophrenia spectrum disorder: An empirical-phenomenological study. *European Archives of Psychiatry and Clinical Neuroscience*, *274*(6), 1405–1415. Scopus. https://doi.org/10.1007/s00406-023-01609-7

Stopa, L., Denton, R., Wingfield, M., & Taylor, K. N. (2013). The fear of others: A qualitative analysis of interpersonal threat in social phobia and paranoia. *Behavioural and Cognitive Psychotherapy*, *41*(2), 188–209. Scopus. https://doi.org/10.1017/S1352465812000422

Taylor, C. D. J., Haddock, G., Speer, S., & Bee, P. E. (2020). Characterizing core beliefs in psychosis: A qualitative study. *Behavioural and Cognitive Psychotherapy*, *48*(1), 67–81. Scopus. https://doi.org/10.1017/S1352465819000274

Toh, W. L., Thomas, N., Hollander, Y., & Rossell, S. L. (2020). On the phenomenology of auditory verbal hallucinations in affective and non-affective psychosis. *Psychiatry Research*, *290*. Scopus. https://doi.org/10.1016/j.psychres.2020.113147

Toh, W. L., Thomas, N., & Rossell, S. L. (2024). The Phenomenology of Visual and Other Nonauditory Hallucinations in Affective and Nonaffective Psychosis: A Mixed Methods Analysis. *Journal of Nervous and Mental Disease*, *212*(4), 205–212. Scopus. https://doi.org/10.1097/NMD.0000000000001750

Topor, A., & Di Girolamo, S. (2010). ‘You have to be careful who you talk to and what you say …’—On psychosis and making rational choices. *Journal of Mental Health (Abingdon, England)*, *19*(6), 553–561. https://doi.org/10.3109/09638237.2010.520366

Upthegrove, R., Ives, J., Broome, M. R., Caldwell, K., Wood, S. J., & Oyebode, F. (2016). Auditory verbal hallucinations in first-episode psychosis: A phenomenological investigation. *BJPsych Open*, *2*(1), 88–95. Scopus. https://doi.org/10.1192/bjpo.bp.115.002303

Wahyuni, S. E., Nasution, M. L., & Daulay, W. (2021). The patient experiences hallucinations with schizophrenia. *Open Access Macedonian Journal of Medical Sciences*, *9*(T3), 112–115. Scopus. https://doi.org/10.3889/oamjms.2021.6350

Wood, H., Cupitt, C., & Lavender, T. (2015). The Experience of Cognitive Impairment in People with Psychosis. *Clinical Psychology & Psychotherapy*, *22*(3), 193–207. https://doi.org/10.1002/cpp.1878

Xanthopoulou, P. D., Mbanu, J., Chevalier, A., Webber, M., & Giacco, D. (2022). Social Isolation and Psychosis: Perspectives from People with Psychosis, Family Caregivers and Mental Health Professionals. *Community Mental Health Journal*. Scopus. https://doi.org/10.1007/s10597-022-00941-x

Yttri, J.-E., Urfer-Parnas, A., & Parnas, J. (2020). Auditory Verbal Hallucinations in Schizophrenia: Mode of Onset and Disclosure. *Journal of Nervous and Mental Disease*, *208*(9), 689–693. Scopus. https://doi.org/10.1097/NMD.0000000000001179
